# Tiny giants of gene regulation: experimental strategies for microRNA functional studies

**DOI:** 10.1002/wdev.223

**Published:** 2016-03-07

**Authors:** Bruno R. Steinkraus, Markus Toegel, Tudor A. Fulga

**Affiliations:** ^1^ Weatherall Institute of Molecular MedicineRadcliffe Department of Medicine, University of OxfordOxfordUK

## Abstract

The discovery over two decades ago of short regulatory microRNAs (miRNAs) has led to the inception of a vast biomedical research field dedicated to understanding these powerful orchestrators of gene expression. Here we aim to provide a comprehensive overview of the methods and techniques underpinning the experimental pipeline employed for exploratory miRNA studies in animals. Some of the greatest challenges in this field have been uncovering the identity of miRNA–target interactions and deciphering their significance with regard to particular physiological or pathological processes. These endeavors relied almost exclusively on the development of powerful research tools encompassing novel bioinformatics pipelines, high‐throughput target identification platforms, and functional target validation methodologies. Thus, in an unparalleled manner, the biomedical technology revolution unceasingly enhanced and refined our ability to dissect miRNA regulatory networks and understand their roles in vivo in the context of cells and organisms. Recurring motifs of target recognition have led to the creation of a large number of multifactorial bioinformatics analysis platforms, which have proved instrumental in guiding experimental miRNA studies. Subsequently, the need for discovery of miRNA–target binding events in vivo drove the emergence of a slew of high‐throughput multiplex strategies, which now provide a viable prospect for elucidating genome‐wide miRNA–target binding maps in a variety of cell types and tissues. Finally, deciphering the functional relevance of miRNA post‐transcriptional gene silencing under physiological conditions, prompted the evolution of a host of technologies enabling systemic manipulation of miRNA homeostasis as well as high‐precision interference with their direct, endogenous targets. *WIREs Dev Biol* 2016, 5:311–362. doi: 10.1002/wdev.223

For further resources related to this article, please visit the WIREs website.

## INTRODUCTION

MicroRNAs (miRNAs) represent an abundant class of endogenous short noncoding RNAs approximately 22 nucleotides (nt) long, which provide an essential post‐transcriptional regulatory layer of gene expression in development and disease.^1^, ^2^ The first miRNA–target axis was discovered in *C. elegans* in 1993, spurring the search for analogous interactions across the entire kingdom of life.^3^, ^4^ Since then miRNAs have been identified and extensively studied across nearly all clades including viruses, unicellular organisms, plants and metazoans. In mammals, approximately 1–3% of the genome codes for miRNA genes and it is estimated that miRNA response elements (MREs) are encoded in the mature sequences of nearly all coding transcripts.^1^, ^5^ Consequently, miRNAs have been shown to orchestrate vital biological processes, such as developmental timing,^3^, ^4^, ^6^ cell fate determination,^7^ and stem cell maintenance.^8^ Furthermore, miRNAs have been linked to the onset and progression of a large number of human pathological conditions,^9^ including various types of cancer. Notably, miRNAs have been implicated both in carcinogenesis (oncomiRs)^10^ as well as in tumor suppression,^11^ and their unique expression profile has been harnessed to classify certain cancer types.^12^ These features, together with the observation that miRNAs can be secreted and are stable in plasma, make them prominent accessible biomarkers as well as therapeutic targets. Notably, due to their ability to silence gene expression, miRNAs have been hailed as potential therapeutic agents capable of targeting ‘undruggable’ pathways where interfering with pathogenic proteins using small molecule compounds has remained ineffective. As a result, widespread attempts have been made to exploit miRNAs diagnostically and therapeutically, which have led to the development of powerful drugs such as miRavirsen, the first miRNA inhibitor to reach Phase II clinical trials for treatment of hepatitis C infections.^13^ All these advances relied on an in depth understanding of miRNA biology and mechanism of action.

**Figure 1:**
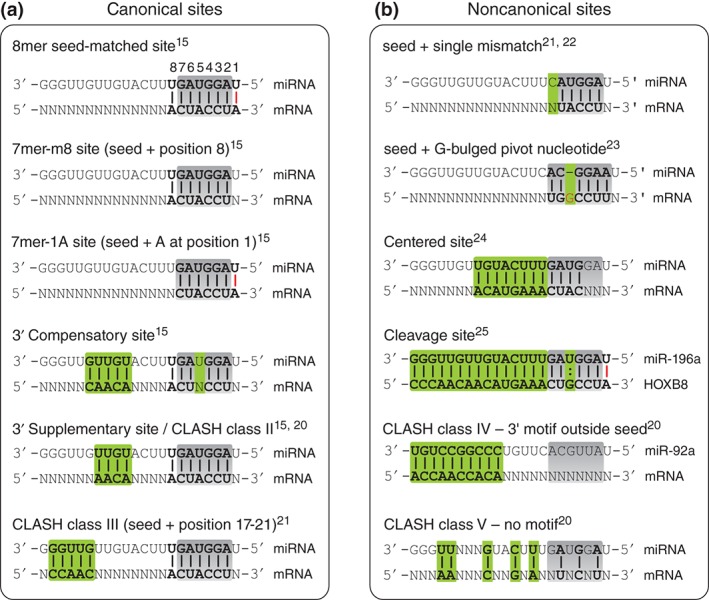
Schematic representation of different MRE types. (a) Canonical sites are defined by perfect complementarity with the miRNA seed sequence. 8mers are matched from position 1–8 and confer the strongest repression. 7mer‐m8 sites are matched at position 8 in addition to position 2–7. 7mer‐1A sites bear a 6mer seed as well as an A‐U pair at position 1. 3′ compensatory sites compensate a G:U wobble (or mismatch) within the seed by complementarity outside the seed. CLASH class II and III are both seed matched but display recurring complementarity at position 13–16 and 17–21, respectively. (b) Noncanonical sites are defined by mismatches within the seed region. Sites with single nt mismatches in the seed were often reported in multiple high‐throughput studies. A G bulged pivot nucleotide was frequently found between position 5 and 6 of the miRNA in Ago‐CLIP datasets. Centered sites display longer consecutive complementarity with only partial involvement of the seed. Cleavage sites possess extensive complementarity leading to slicing of the target. CLASH class IV sites have minimum 9 nt consecutive pairings outside the seed region. CLASH class V are orphan clusters without recurring motifs. *Gray boxes* = *miRNA ‘seed’ region (nucleotides 2–7); green boxes denote characteristic motifs for each class*; *bold = base paired nucleotides; : = G:U wobble; red bar = complementarity at position 1 of the miRNA (unlikely to allow base pairing in vivo since this position is anchored inside Ago)*.

Although null mutants of the first discovered miRNAs uncovered dramatic phenotypes, it subsequently became apparent that in general miRNAs function primarily as molecular rheostats fine‐tuning gene expression and modulating transcriptional noise, rather than acting as binary switches.^14^, ^15^, ^16^ However, the search for *in vivo* biological functions of miRNAs remains a challenging endeavor primarily due to the relatively permissive thermodynamic parameters required for productive binding of miRNAs to their targets.^17^, ^18^, ^19^ Consequently, understanding the physiological role of miRNAs in a cellular context invariably requires an exigent search for their direct targets. At molecular level, although miRNA targeting is governed by stereotypical Watson‐Crick base‐pairing rules, target binding is mediated by relatively promiscuous, incomplete complementarity. This rendered bioinformatics target identification using classical sequence alignment tools ineffective and unreliable. Therefore, substantial effort has gone into deconstructing the molecular logic of MREs. Genomic analyses of miRNA–target interactions revealed strongly conserved complementarity for approximately 6–8 base pairs from position 2 of the miRNA^1^ (Figure 1(a)). This region (nucleotides 2–7 at the 5′ end of the miRNA) has been henceforth termed the ‘seed’ sequence and formed the basis for the development of the first computational miRNA target prediction algorithms. However, heterogeneous configurations have been discovered within this sequence, resulting in varying potency of interaction: 8mer seeds are assumed to be the most potent, followed by 7mer‐m8 (matched at position 8), 7mer‐A1 (adenosine at position 1), and finally 6mers (nucleotides 2–7).^26^ Furthermore, 3′ compensatory sites,^15^ centered sites^24^ and offset 6mers have also been reported (Figure 1(a) and (b)). While seed pairing is still widely recognized as the archetypal determinant factor for miRNA target recognition and binding, the discovery of noncanonical interactions suggests that even more MRE categories exist than originally anticipated, and novel site types continue to emerge^20^, ^21^, ^23^, ^27^, ^28^ (Figure 1(b)). However, the competence of such noncanonical MREs to mediate target repression has recently been challenged and thus remains controversial.^29^ Regardless, these discoveries add another layer of complexity to the quest for in depth characterization of miRNA physiological functions.

In addition to MRE sequence determinants, it has become widely accepted that other intrinsic and extrinsic factors such as MRE secondary structures^30^ and their association with RNA binding proteins (RBPs), can have a considerable impact on miRNA–target interactions (for review see Refs 31, 32). Since most of the coding transcriptome appears to be decorated by proteins,^33^ it is conceivable that certain protein–RNA interactions could enhance miRNA regulation while other can suppress their activity. An example of positive regulation is provided by the *Pumilio* protein family that bind E2F3 and p27 and enhance the effect of miRNA translational repression on these targets.^34^, ^35^ Conversely, in zebrafish germline cells, *dead end 1 (Dnd1)* appears to mask *miR‐430* target sites in *nanos* and *tdrd7* mRNAs, thus reducing its repressive action.^36^ Another well documented example is provided by the HuR (ELAV) family of AU‐rich element (ARE) binding proteins that form dynamic interactions with RNAs and can influence under certain physiological conditions miRNA‐mediated silencing.^37^, ^38^ For example, exposure of Huh7 hepatoma cells to stress induces a HuR‐dependent derepression of *miR‐122* activity on *CAT‐1*, and relocation of the *CAT‐1* mRNA from processing bodies to ribosomes.^37^, ^39^ High‐resolution HuR‐RNA binding maps revealed that HuR sites are frequently present within close proximity of MREs but do not necessarily overlap, alluding to potential widespread HuR‐miRNA functional interactions.^40^, ^41^ Correspondingly, Ago2 genome‐wide binding studies uncovered significantly more frequent miRNA association with RNAs harboring MREs within a 30 nt window of HuR consensus elements.^42^ Supporting a pervasive functional impact of HuR in miRNA‐mediated repression, targets carrying MREs within this 30 nt window were significantly more repressed in HuR mutant cells, while MREs outside this window did not show such an effect, even at high local density.^42^ A recent *in vitro* study proposed that HuR can oligomerize along an RNA and thus physically displace the miRISC complex from the target mRNA, providing a potential mechanistic insight into how HuR proteins compete with miRNAs.^43^ Interestingly, in cervical carcinoma HeLa cells it was reported that HuR is required for *let‐7* mediated repression of *c‐myc*, suggesting a cooperative rather than an antagonistic effect.^38^

Other factors that have been reported to interfere with or provide additional layers of regulation to miRNA‐activity include, ARE,^44^, ^45^ poly‐A binding proteins,^46^, ^47^ and the tripartite motif TRIM‐NHL class of proteins.^48^, ^49^ With regard to the latter, the *C. elegans* NHL‐2 was shown to associate with both processing body components as well as ALG‐1/2 and AIN‐1, the nematode homologues of Ago and GW182.^48^ This interaction was proposed to promote the action of *let‐7* and *lsy‐6*, and incidentally confer robustness onto vital development transitions.^48^ In mice, it was proposed that TRIM32 associates with Ago1 and augments, in particular but not exclusively, the role of *let‐7a* in asymmetric cell division during neuronal differentiation.^49^

While miRNAs provide the specificity code for determining which mature transcripts will be targeted for regulation, their repressive activity is mediated by a multifactorial effector protein complex generically termed, *via* analogy to RNAi, the *miRNA induced silencing complex* (miRISC). The core functional component of miRISC is represented by members of the highly conserved Argonaute (Ago) family of proteins, which directly contact both the miRNAs and their cognate target RNAs.^50^ Unlike the Ago proteins associated with short interfering RNAs (siRNAs) or plant miRNAs, in animals, miRNA‐directed Ago does not mediated cleavage (slicing) of their bound RNA targets except in extremely rare cases,^25^ or when participating in Dicer‐independent miRNA biogenesis.^51^, ^52^ Instead, the dominant effect of animal miRISC binding to target mRNAs appears to be transcript destabilization, which occurs as a consequence of deadenylation followed by decapping and 5′→3′ RNA decay.^53^, ^54^, ^55^, ^56^, ^57^, ^58^, ^59^ However, it has been proposed that this fateful and irreversible miRNA‐mediated effect on cellular mRNAs is often preceded by transient translational repression,^60^, ^61^, ^62^ which sometimes may also occur independent of mRNA degradation.^4^, ^60^, ^63^ Multiple models have been proposed to explain miRNA‐mediated translational repression, including abrogation of translation initiation and blocking of elongation.^64^, ^65^ Nonetheless, the molecular mechanism underlying miRNA‐mediated gene regulation remains a topic of active investigation, and it is conceivable that a variety of different scenarios will prove plausible depending on the biological context under investigation.

The biogenesis, regulation, and operational modes of miRNAs have been extensively covered by a large number of high quality reviews.^15^, ^50^, ^66^, ^67^, ^68^ The scope of this review is to provide an overview and evaluate current state‐of‐the‐art technologies for miRNA research in animals, as well as guide the researcher in navigating and deploying in a step‐wise fashion the multidimensional miRNA toolkit. The first section provides an introduction to bioinformatics miRNA target prediction algorithms. The second part is focused on experimental target identification with special emphasis on high‐throughput platforms and large‐scale studies. The third and final section covers in detail the technologies underlying functional miRNA studies.

## *IN SILICO* miRNA TARGET PREDICTION

The absence of a gold standard for identifying direct miRNA–target binding events led to the conception of bioinformatics algorithms to help navigate the vast number of putative miRNA–target interactions that can take place in a cell. Since the first *in silico* tool was published in 2003, a slew of miRNA target prediction algorithms have been developed and subsequently evolved, making the subject of numerous in depth review articles.^2^, ^69^, ^70^, ^71^, ^72^, ^73^, ^74^, ^75^, ^76^, ^77^, ^78^, ^79^, ^80^, ^81^ In a relatively oversimplified view, the most commonly used algorithms could be broadly divided into *filtering* and *machine learning* (ML) approaches,^79^ depending on their *modus operandi*. *Filtering approaches* are in essence based on defined features against which a dataset is screened, and matches are classified as putative targets. The screening criteria are mainly based on experimentally defined features that frequently include the architecture of the miRNA seed match, the evolutionary conservation of the MRE, as well as the thermodynamic parameters underpinning each putative miRNA–target pair. *ML approaches* rely on training a classifier with positive (true target) and negative (false target) datasets and then applying it to a new dataset. As a general rule, it is advisable to consider both strategies. Filtering approaches have higher interpretability but suffer from relatively low specificity (false positives) and reduced sensitivity (false negatives).^82^ In contrast, ML approaches provide in principle superior specificity, but they tend to be more difficult to implement into routine experimental pipelines.

In the following sections we provide a brief overview of both filtering and ML algorithms, for the experimental biologist (summarized in Table 1). We also discuss some of their advantages and disadvantages, and offer suggestions regarding the appropriate algorithm choice for various applications. Finally, we mention some limitations of current bioinformatics tools, and evaluate the potential of considering the MRE biological context in the development of next‐generation *in silico* approaches.

**Table 1:**
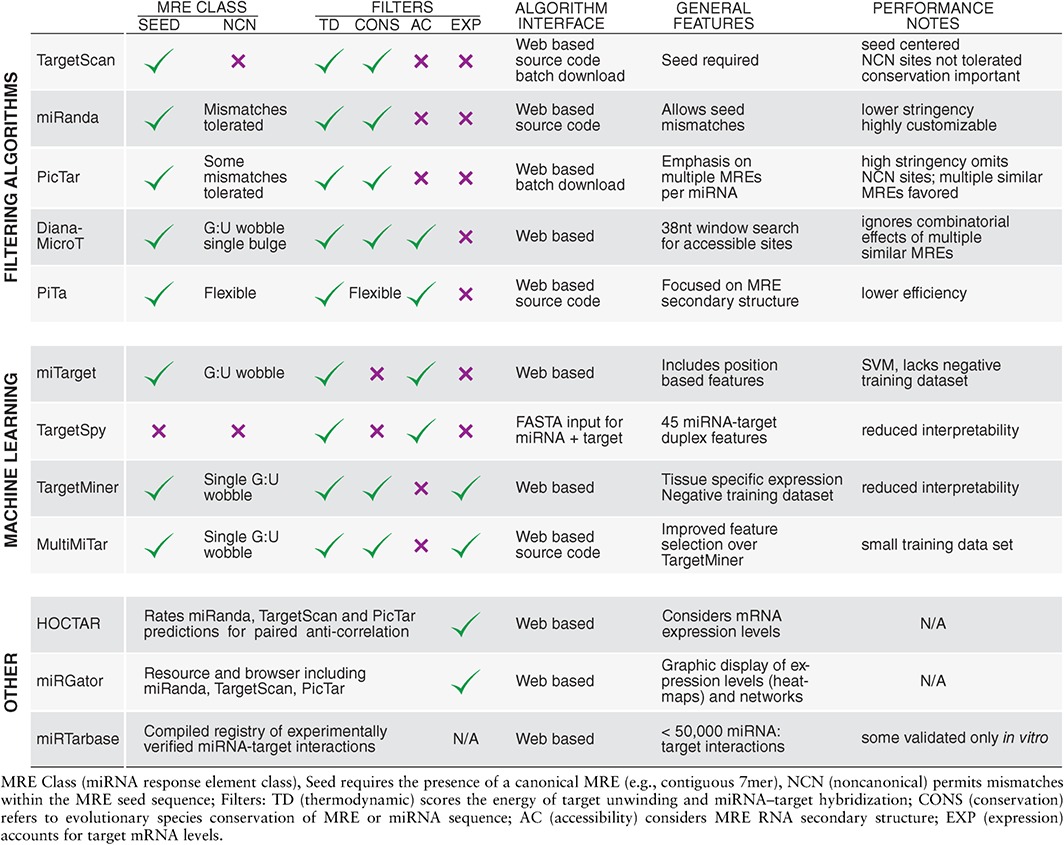
Overview of Bioinformatics Tools for miRNA Target Predictions

## TARGET SITE INTRINSIC ALGORITHMS

Target site intrinsic algorithms take into account primarily the molecular architecture of the MRE, such as degree of base pairing and thermodynamic parameters, and the evolutionary conservation of the putative miRNA–target interaction. In general, to find an adequate algorithm for a specific application it is important to consider the contribution of each of these parameters. For example, if the miRNA under investigation is not conserved across species, filtering for MRE conservation may be counterproductive. Likewise, the tolerance of the algorithm toward base‐pairing mismatches within the seed region of the MRE (and the ability to customize this parameter) can impact both positively and negatively the discovery of putative miRNA–target interactions. For example, permissive algorithms will allow the detection of functionally validated imperfect seed sites (G:U wobbles, G‐bulge sites, etc.), at the expense of possibly increasing the rate of false positive hits.^23^ Another parameter that should be considered when predicting miRNA–target interactions is the range of genomic elements considered by the search algorithm. Although it is broadly accepted that most effective MREs are located within the 3′UTRs, it has been reported that a host of miRNAs can successfully and often with the same frequency target ORFs and the 5′UTRs.^83^, ^84^ Therefore, for a comprehensive analysis, it may be necessary to choose computational platforms that allow predictions outside of 3′UTRs.

### Filtering Methods

Since it was established that the most important determinant for canonical miRNA targeting appear to be the seed region,^1^, ^85^ the earliest developed algorithms filtered putative target interactions for seed complementarity.^86^, ^87^, ^88^ The Bartel/Burge labs and separately the Marks lab were among the first to develop what arguably even today remain two of the most commonly used and reliable interactive tools for miRNA target prediction: TargetScan and miRanda (**microrna**.**org**). The **TargetScan** interactive web‐interface is accessible at www.targetscan.org and it supports both miRNA and target gene queries in five species: human, mouse, zebrafish, *Drosophila* and *C. elegans*.^86^ In essence, the algorithm searches for consecutive complementarity to nucleotides 2–8 of the miRNA 5′ end, and computes the free energy of the resulting miRNA‐expanded seed region duplex employing the RNAfold package.^89^ Over time, the algorithm has evolved to include a range of search parameters, including prediction of poorly conserved MREs and miRNAs, as well as tolerance to seed mismatches coupled with compensatory conserved pairing outside the seed. Predicted interactions are scored using at least six parameters, and filtered for conservation against a broad spectrum of vertebrate and invertebrate species. The scores are calculated based on both MRE intrinsic parameters (site‐type, 3′ pairing) and the MRE context (AU content, MRE position within the UTR, target site abundance, seed‐pairing stability).^90^ Furthermore, the recent introduction of TargetScan ORF permits scanning against open reading frames in addition to 3′UTR sites. Although a web‐based search for ORF MREs is only available for *Drosophila* at the moment, the computed ORF sites for the mouse and human genomes are available for download. Notably, the TargetScan source code is freely accessible and can be downloaded as a Perl script, enabling the analysis of any user‐defined sequence or custom data set.

**miRanda** is an independently developed web‐accessible miRNA target prediction resource, operating with slightly less stringent parameters and thus generally predicting more putative sites.^87^ Similar to TargetScan, this platform searches for seed‐biased complementarity, takes into consideration the free energy of miRNA–target duplex formation, and uses the phylogenetic hidden Markov model of the PhastCons algorithm to calculate a conservation score across several vertebrate species. Target predictions are also based on a set of pre‐established biological rules, and are available for all mature miRNAs in human, mouse, rat, *Drosophila*, and *C. elegans*. An important upgrade of this resource was made with the incorporation of mirSVR, a regression model which takes into account both sequence and context features of the miRNA–target duplex.^91^ A host of parameters are considered by this model, including seed pairing and complementarity at the miRNA 3′ end, AU composition in the vicinity of MREs, secondary structure predictions spanning the target sites, length of the 3′UTR, and the relative position of the MRE within the 3′UTR. The integration of miRanda target prediction, mirSVR score and phastCons evolutionary conservation, resulted in the release of a unified comprehensive web‐interface (microrna.org) which provides not only information regarding putative MREs but also the likelihood of target downregulation. Furthermore, this integrated approach enables the prediction of noncanonical sites containing mismatches or G:U wobbles in the seed region, without apparently increasing the number of false positive interactions. Finally, the miRanda code is also available as an open source license, allowing the input of any user‐defined miRNA and target sequence as well as a largely customizable set of search parameters.

A number of other algorithms provide various degrees of stringency as well as additional features. For example, PicTar and **PicTar 2.0** (**P**robabilistic **i**dentification of **c**ombinations of **Tar**get sites) is another seed‐based algorithm, which scans the 3′ UTRs of genes for approximately 7 nucleotides complementarity near the 5′ end of the miRNA.^92^, ^93^ Similar to TargetScan and miRanda, it calculates free energy scores and filters for conservation of the target site in several species. However, PicTar adds an additional layer of stringency by strongly emphasizing multiple miRNA target sites within the same mRNA target.

An optimal miRNA target prediction algorithm possesses high specificity (low number of false positives) as well as high sensitivity (low number of false negatives). Although filtering for evolutionary conservation of the predicted MRE may reduce the number of false positive hits, it may inadvertently inflate the number of false negative results. This phenomenon is mainly observed when the conservation parameter is not restricted only to the MRE sequence in isolation, but extends to positional conservation, in particular when the target site is in the ORF of a gene.^72^, ^80^ Therefore, the pre‐alignment of orthologous sequences from various species may present a problem as a functional site may be conserved but not in position in a forced multiple sequence alignment.^78^ Based on these considerations, it was proposed that approximately one third of mammalian target sites are not identified by alignment because they are not positionally conserved.^70^ Therefore, although a powerful feature, conservation analysis may under certain circumstances restrict *in silico* analyses.

To overcome some of these limitations, various algorithms have been developed to take into account additional features, such as target site availability for binding. For example, while still using pre‐aligned blocks for conservation analysis, **DIANA‐microT** is distinguished by the fact that it screens targets for binding availability using a 38‐nucleotide window,^94^, ^95^ and the minimum binding energy is calculated for each possible target interaction. In contrast to approaches that favor multiple target sites (e.g., PicTar), this algorithm does not score multiplicity of target sites in the same transcript. A notable feature of DIANA‐microT is that the web interface allows input of custom user‐defined miRNA sequences. Finally, in the most recent version, DIANA‐microT‐CSD, the algorithm also includes predictions in mRNA coding regions.^96^, ^97^ Another interesting platform is **PITA** (**P**robability of **I**nteraction by **T**arget **A**ccessibility).^30^ PITA also allows user‐defined sequences to be uploaded directly through the web interface (both miRNA and target RNA), and the conservation filtering parameters are fully customizable. However, PITA is neither biased toward seed‐based interactions, nor does it require cross‐species conservation, therefore making it particularly useful for the prediction of noncanonical target sites. One of the distinctive features of the PITA algorithm is that it calculates the free energy required to melt the target RNA secondary structure in order to render it accessible to hybridize to the miRNA, as well as the free energy gained by miRNA binding to the MRE. Both DIANA‐microT and PITA bring an additional layer of flexibility to miRNA target prediction algorithms. However, defining the optimal search window is not immediately intuitive, and this parameter can significantly influence the accuracy of the predictions, resulting in a large number of false positive hits.^82^

### ML Approaches

The advent of experimental strategies for high‐throughput identification of miRNA–target binding events in living cells (see next sections) demanded a re‐evaluation of the seed‐targeting dogma. Over the past few years it has become apparent that a considerable number of miRNA–target interactions do not obey the aforementioned rules, but rather exhibit relatively heterogeneous binding patterns.^20^ As increasing numbers of miRNA–target interactions have been validated and recorded in registries like **miRTarBase** and **miRecords**,^98^, ^99^ ML approaches joined the pursuit and added a new dimension to the development of miRNA target prediction algorithms. Although several ML platforms have been developed almost a decade ago, their implementation in routine miRNA research pipelines has met with relatively limited success. A brief overview of the most commonly used ML platforms is provided below.

**miTarget** is a support vector machine (SVM) based ML strategy which relies on multiple miRNA–target features, including secondary structure, thermodynamic parameters, and positional data.^100^ Taking advantage of the RNAfold program from the Vienna RNA Package, the algorithm calculates the free energy scores of three MRE parts: the seed region, 3′ segment, and the entire miRNA–target alignment. The program is trained on an existing microarray dataset, compares the computed vectors of putative interactions to true and false targets, and predictions are filtered for functional relevance by gene ontology (GO) analysis. **TargetSpy** was developed in 2009 to search for miRNA target sites independently of seed match or conservation.^101^ The algorithm automatically selects experimentally defined features and has been estimated to predict from 26 to as many as 112 noncanonical sites for each miRNA that go undetected by other algorithms. Although the ML component was only trained on mouse targets, its performance was also tested on human and *Drosophila* miRNAs. **TargetMiner** is one of the first SVM based classifiers to systematically incorporate experimentally validated negative interactions from high‐throughput studies as a training dataset.^102^ This notable improvement reduced the chance of including true targets in the negative training set, which may have occurred when randomized sequences were used for this purpose. Its improved version, **MultiMiTar**, now employs an enhanced feature selection algorithm for positive interactions and generates a ranked list of putative miRNA targets.^103^

An inherent shortcoming of ML implementations is that their performance relies heavily on the quality of the training dataset. However, the continuous development and evolution of high‐throughput target identification assays as well as the growing number of validated functional miRNA–target interactions, are increasing the confidence and quality of training datasets. As a result, this is likely to facilitate in the near future the development of more powerful and accurate ML miRNA target prediction algorithms.

Although a key determinant in advancing miRNA research, the continuous expansion of bioinformatics platforms for target prediction inevitably created a dilemma: how does one choose the most reliable algorithm amidst all available options? To simplify this process, efforts have been directed toward the development of integrated platforms which can automatically parse data from multiple target prediction algorithms as well as information on experimentally validated miRNA–target interactions. One notable resource is the recently updated **miRwalk 2.0**, a powerful multilayered database, which integrates predictions from 13 different tools including TargetScan, miRanda, PITA, PicTar, and many others, and incorporates experimentally validated target interactions.^104^, ^105^ The searchable web interface, which includes a ‘predicted target module’ and ‘validated‐target module’, allows a multipronged customizable data visualization of all putative MREs (full gene length) across fifteen different species. By mining a host of other databases, miRwalk 2.0 provides background information on both miRNAs and their predicted targets regarding ontology, epigenomic profiles, pathway analysis, phenotype, genotype, SNPs, functional networks, and relevant publications. Finally, a new feature now enables users to sample putative miRNA‐lncRNA interactions in addition to mRNA target genes. In particular for high‐throughput studies, this type of ‘hub resources’ may prove extremely useful in streamlining the bioinformatics workflow underlying miRNA research.

## THE NEXT FRONTIER: EMERGING REGULATORY CONTEXT

### Spatial and Temporal Co‐Expression

An obvious prerequisite for functional target regulation is that both the miRNA and the target are co‐expressed in the same cell and in overlapping subcellular compartments. Consequently, the binding kinetics of miRNA–target interactions are dependent among other physicochemical parameters on the local concentration of a miRNA and its targets. Although this factor is likely to influence thermodynamic computation, it is only approximated when considered by target prediction algorithms. However, with the advent of next‐generation sequencing, expression data could now be easily incorporated in miRNA studies, by paired dual profiling of both total RNA and miRNAs from the same samples. Assuming that mRNA destabilization and decay is the dominant consequences of miRNA targeting, this approach could add a new layer of confidence in prediction algorithms. However, it would be less informative for situations where miRNA binding only causes translational repression of their targets. Three main routes have been proposed for integrating paired expression data with target prediction algorithms: correlation based, linear mode approach and Bayesian network oriented (reviewed by Naifang Su et al.).^106^ For example, **HOCTAR** is a correlation‐based approach that incorporates predictions from TargetScan, miRanda and PicTar, and ranks these according to anti‐correlation of expression levels.^107^ **miRGator** is a popular alternative, which provides correlation‐based user friendly heat‐maps.^108^ Generally, due to the variable degradation kinetics of miRNA targets, it is likely that correlation‐based methods may be more useful for excluding low confidence targets rather than increasing the confidence of predicting *bona fide* interactions.

### Stoichiometry and Threshold Levels

In addition to correlation‐based filters, the analysis of cellular miRNA and target levels can bring a new dimension to functional studies and confer further insight into the development of target prediction algorithms. For example, Mukherji et al. investigated the effect of target mRNA abundance on miRNA‐mediated repression at single cell level.^109^ This analysis revealed that miRNAs strongly repress protein production below a certain level of transcript abundance. Under this threshold, the effect appears to be universal across all targets independent of their expression, as long as the entire target pool did not reach saturation levels for the miRNA. However, if any target abundance reaches a level sufficient to titrate away its cognate miRNA, a ‘sponging’ effect will occur resulting in derepression across all cellular targets. Similarly, the levels of miRNA expression also appear to significantly impact target repression activity. A large‐scale functional study proposed that only the most highly expressed miRNAs (top 40% of the cellular miRNome) appear to display detectable target suppression activity, as revealed by a multiplex sensor assay.^110^ This raises the possibility of a ‘functional threshold’ under which miRNAs are effectively inactive and could thus be excluded from further investigation, which concomitantly would have the potential to strongly reduce false positive predictions. Based on these considerations, it would make sense to incorporate expression levels and miRNA–target stoichiometry parameters into existing target prediction algorithms.

### RNA Binding Proteins

Another layer of complexity in miRNA biology, which directly impacts the accuracy of target prediction algorithms, stems from the propensity of cellular RNAs to interact with a host of RBPs. A better understanding of protein‐binding motifs encoded within mRNAs will undoubtedly engender new insights into miRNA function and possibly improve prediction tools by filtering out putative MREs that are unlikely to be accessible for miRNA binding. The Hentze group has recently developed a powerful strategy to identify all binding proteins associated with cellular RNAs bearing a poly‐A tail.^33^, ^111^ Harnessing this information and including it into prediction algorithms will require accurate mapping and classification of RNA protein‐binding sequence determinants, as well as an in depth understanding of their impact on miRNA activity. Currently, the number of miRNA target prediction programs that take into consideration RBP motifs is very limited. One of the first computational tools to fulfill this criterion is **MREdictor**, an algorithm that evaluates the impact of target site accessibility and the presence of Pumilio recognition element (PRE) motifs in the proximity of MREs.^112^ Interestingly, analysis of functionally validated targets revealed that PRE motifs appear to be preferentially located in the vicinity of inaccessible MREs, suggesting that they may generally enhance or facilitate miRNA‐mediated repression. Since novel RBPs are constantly discovered, the integration of RNA motif search tools such as **RegRNA 2.0** 113 into target prediction algorithms will be important to automatically evaluate putative miRNA targets for the presence of overlapping RNA regulatory elements.

### 3′UTR Isoforms and Alternative Poly‐Adenylation

A study comparing the predictive power of TargetScan against two reference databases, UCSC (TargetScan default source) and ENSEMBLE (miRanda default source), aimed to establish whether varying transcript annotations can have an impact on target predictions.^114^ This analysis revealed an astonishingly low concordance rate of only 47% between the two datasets, demonstrating that in addition to intrinsic computational variables, input transcript annotation can significantly promote bias in miRNA target prediction algorithms.

Even if annotations were standardized, inherent 3′UTR genetic heterogeneity presents perhaps an even greater challenge for miRNA research. An in‐depth bioinformatics analysis revealed that as many as two thirds of predicted miRNA target genes have alternative 3′UTRs, and 40% of all predicted MREs are encoded within alternative UTRs.^115^ This can have profound consequences for *in silico* predictions, since most algorithms do not accommodate more than one 3′UTR isoform. For example, the mammalian version of TargetScan, only takes into account the longest 3′UTR isoform of coding transcripts. The predominant molecular mechanism promoting generation of 3′UTR isoforms is alternative cleavage and poly‐adenylation (APA).^116^ Many transcripts bear multiple 3′UTR poly‐adenylation sites, which can be engaged under various circumstances, resulting in shorter or longer isoforms.^116^ Although MREs upstream of the first poly(A) site are never affected by APA, those downstream of this site may get eliminated from mature transcripts depending on whether a proximal or distal poly(A) site is used. This process has a significant regulatory potential which can be harnessed by developmental programs, as it was recently demonstrated in zebrafish.^117^ However, the same mechanisms may also underlie disease pathogenesis as reported for the proto‐oncogene IGF2BP1/IMP‐1, which promotes tumorigenesis by escaping miRNA‐mediated repression through alternative poly‐adenylation.^118^

To decipher the widespread impact of cellular environment including APA on miRNA‐mediated repression, a recent study analyzed the effect of deploying the same miRNAs (*miR‐124* and *miR‐155*) in different cell lines and tissues.^119^ Transcriptome‐wide RNAseq analysis revealed that by *en large* predicted targets appeared to be consistently regulated irrespective of cellular context. Interestingly, mRNA targets not displaying convergent repression were frequently subject to APA. Based on these results, a new parameter termed *affected isoform ratio* (AIR) was defined, which represents the fraction of transcript isoforms bearing a specific MRE. Indeed, plotting AIR against mean repression values revealed a significant correlation between these metrics. Furthermore, integration of a weighted AIR factor (termed the wContext+ score) into the linear regression model of TargetScan, improved the overall performance of the algorithm by approximately 50%.^119^ These results suggest that isoform information represents an important metric and should be considered for improving the power of miRNA target prediction algorithms in the future. This endeavor will be facilitated by the advent of poly(A)‐position profiling technologies (3p‐seq), which are substantially improving the accuracy of 3′UTR annotations in an increasing number of cell types and tissues.

## EXPERIMENTAL miRNA TARGET IDENTIFICATION

Although advances in bioinformatics algorithms have the potential to increase the confidence of miRNA target predictions, the reliability of most common algorithms (miRanda, PITA, and TargetScan) still display a relatively high false positive (46–63%) and false negative (44–82%) rate.^82^, ^120^ Therefore, the experimental identification of physiological targets remains one of the crucial steps in miRNA research which is reflected in the multitude of studies and reviews on this subject.^2^, ^69^, ^73^, ^80^, ^121^, ^122^, ^123^, ^124^, ^125^, ^126^, ^127^, ^128^, ^129^, ^130^, ^131^, ^132^, ^133^, ^134^, ^135^, ^136^, ^137^, ^138^, ^139^, ^140^, ^141^ Historically, miRNA–target interactions have been inferred from genetic approaches, foremost in *C. elegans* (reviewed in Ref 133). Mutations in genes that could counteract phenotypes induced by the loss of a miRNA were considered potential candidates for direct interactions. Although still valid, genetic screens are not widely used any more mostly because they are laborious and not easily amenable to all model organisms. Additionally, many miRNAs do not appear to cause any detectable phenotypes in *C. elegans* 142 or mouse,^143^ which is an essential prerequisite for conducting a reverse genetic screen. Consequently, alternative methods for miRNA target identification evolved to assess the physical interactions between miRNAs and their targets. These can either exploit changes in target expression upon miRNA loss‐of‐function or gain‐of‐function or the direct interaction between miRNAs and their targets in the form of binding data (summarized in Tables 2 and 3). These methods are much faster than genetic screens and are also suitable for high‐throughput and genome‐wide approaches.

**Table 2:**
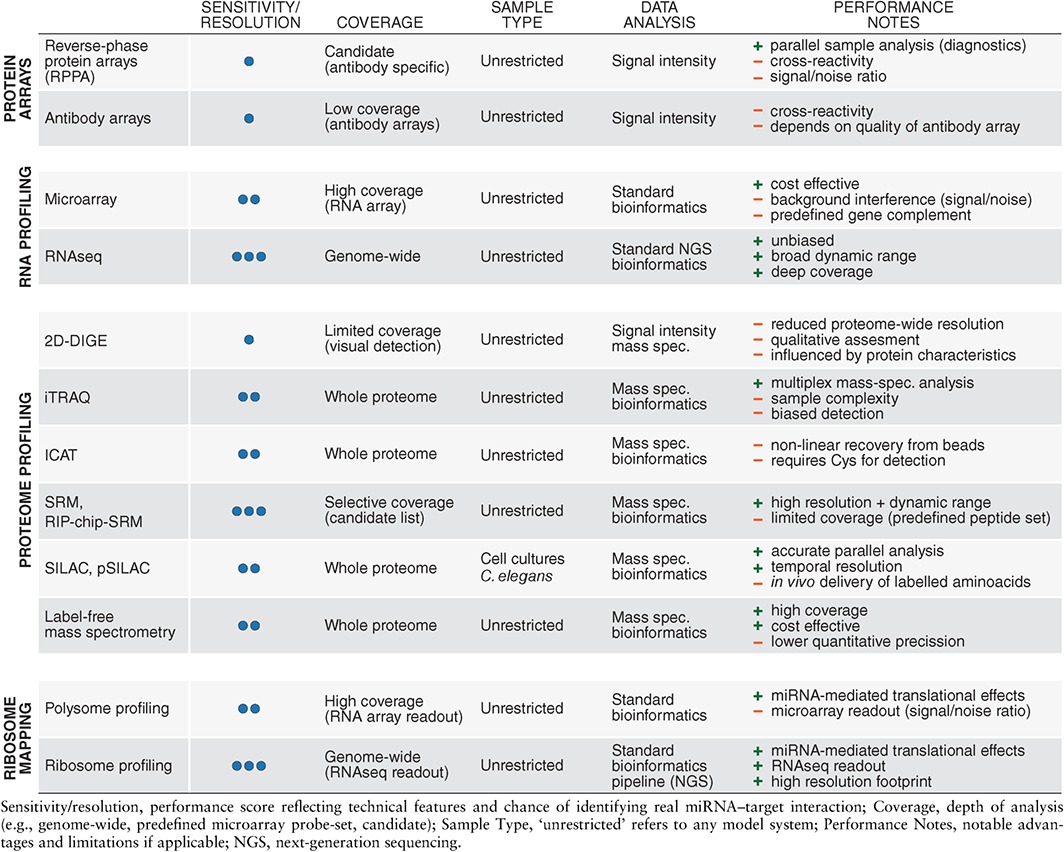
Profiling‐Based Strategies for miRNA Target Identification

**Table 3:**
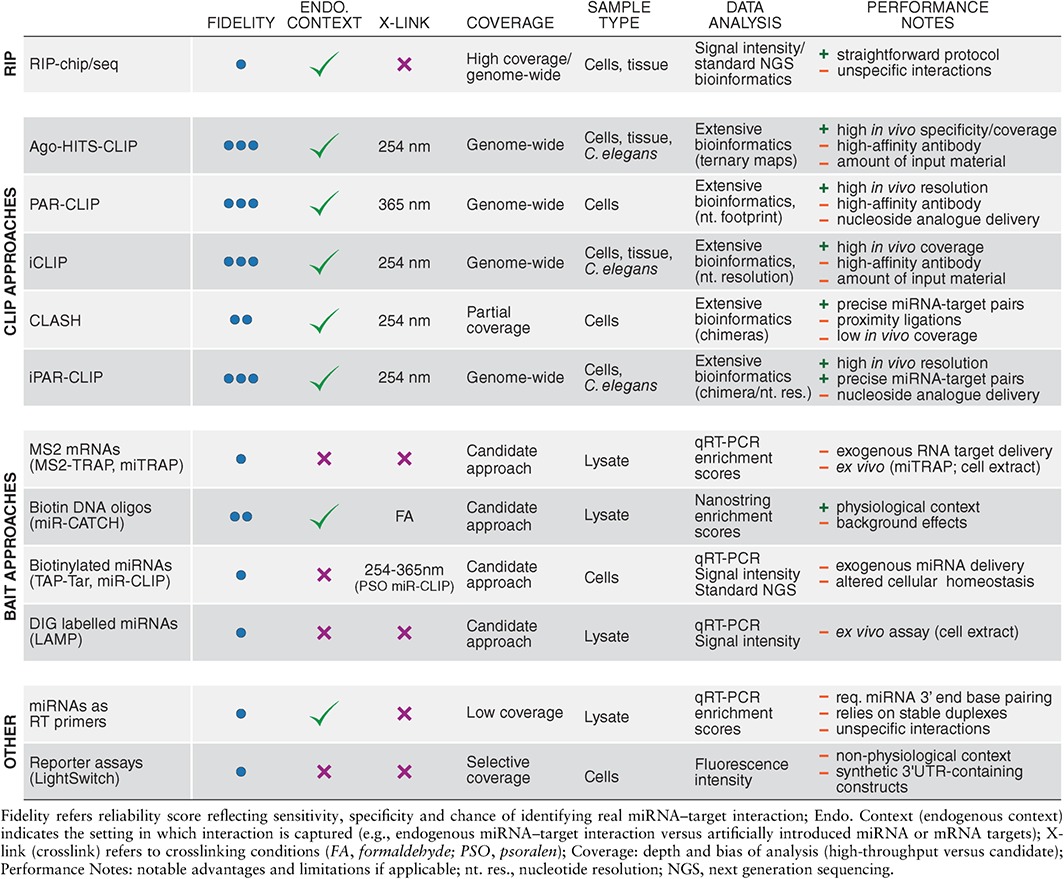
Capture‐based Technologies for Detection of Direct miRNA–Target Binding Events

## PROFILING‐BASED APPROACHES

For small‐scale studies, the expression levels of potential miRNA targets can be analyzed by *in situ* hybridization, qRT‐PCR, Northern blot, Western blot or protein arrays. **Reverse‐phase protein arrays (RPPA)** offer the possibility to investigate a large number of biological samples simultaneously. The arrays are probed with different antibodies in combination with a biotin‐streptavidin‐based detection system to quantify candidate proteins in the samples. Owing to its high‐throughput potential, RPPA is frequently used in clinical diagnostic. This method was used to identify miRNA–target pairs in cartilage samples from patients suffering from osteoarthritis.^144^ By probing the samples with 214 different antibodies against proteins expressed in cartilage, 76 differentially expressed proteins were detected. Potential physiological targets were further defined based on inverse correlation with miRNA expression data and *in silico* predictions.^144^ A closely related platform that has also been adapted to miRNA research is the antibody‐based array technology. Similar to RPPA, **antibody arrays** are incubated with total protein fractions isolated from cell lysates and subsequently exposed to a two‐step detection system consisting of a biotinylated antibody and fluorophore‐conjugated streptavidin. A recent study employed an array containing 71 antibodies against human receptor tyrosine kinases (RTKs), to identify seven RTKs whose signal was altered upon *miR‐206* mimic expression in A549 cells.^145^ A potential direct target of *miR‐206* regulating the most repressed RTK was then proposed based on *in silico* predictions. Although the array was not designed to identify direct miRNA targets, this technique has the potential to be used for miRNA target identification within a defined set of genes. Beyond that, this technology has great value as a molecular diagnostic tool.

In contrast to small‐scale approaches, genome‐wide studies aim to assess the effect of aberrant miRNA expression on a global scale, employing either transcriptome profiling (microarray and RNAseq), proteome profiling (2D‐DIGE, SILAC, iTRAQ, and ICAT), or translational profiling. Conceptually, all these strategies rely on comparative analysis of endogenous gene expression either between different cellular states (e.g., healthy versus disease cells or tissues), and/or under artificially altered miRNA homeostasis (systemic overexpression or inhibition of candidate miRNAs).

### Transcriptome Profiling

**Figure 2:**
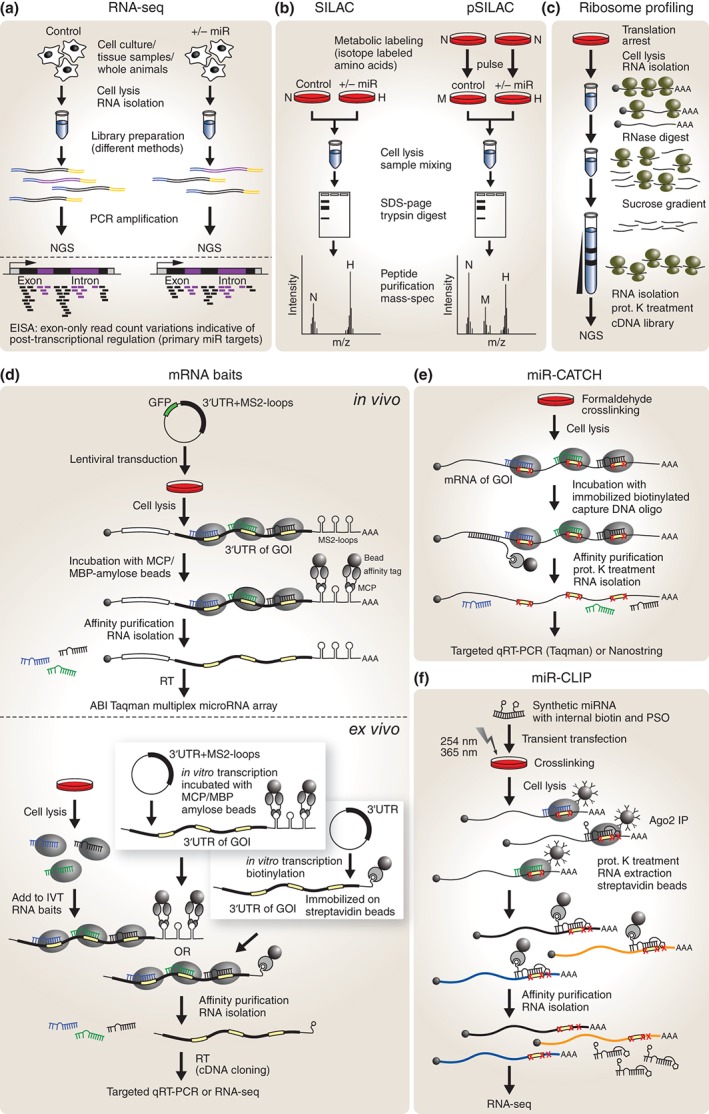
Profiling and pull‐down‐based miRNA target identification techniques. (a) RNAseq yields short sequencing reads from all transcribed genes including 5′UTRs, exons, introns, and 3′UTRs. Intron‐exon split analysis (EISA) has the potential to distinguish between primary and secondary miRNA targets based on intron read counts differences. (b) In SILAC all proteins of the experimental condition are labeled with a heavy (H) isotope version while all proteins of control cells contain a normal (N) isotope version. The ratio between isotope versions indicates differential expression of proteins. In pSILAC a medium (M) and a heavy (H) isotope version are added to the control and experimental condition (=pulse) and differences in newly synthesized proteins are quantified. (c) Ribosome profiling yields all transcripts that are bound by ribosomes and the position of each ribosome with nucleotide resolution. (d) mRNA baits consist of a 3′UTR from the gene of interest (GOI) and a tag (M2‐loops or biotin) that allows for pull‐down via bead coupled protein moieties (MCP or streptavidin). Copurified miRNAs are analyzed by targeted qRT‐PCR or RNAseq. Transduction of the mRNA bait enables association of bait and miRNAs prior to cell lysis *in vivo*, while *in vitro* transcribed baits rely on proper target recognition *ex vivo* after cell lysis. (e) In mir‐CATCH miRNA–target complexes are crosslinked *in vivo* and the mRNA of interest is affinity purified via antisense capture oligonucleotides (oligo). After crosslinking reversal, copurified miRNAs are analyzed by targeted qRT‐PCR or nanostring. (f) In miR‐CLIP UV crosslinking is enhanced *via* a psoralen group. To reduce background a two‐step purification protocol is performed prior to quantification of copurified RNAs by RNAseq. MCP, MS2 coat protein; MBP, maltose binding protein; RT, reverse transcription; prot. K, proteinase K; SDS‐PAGE, sodium dodecyl sulfate polyacrylamide gel electrophoresis.

The original strategies for large‐scale comparative gene expression analysis relied on **microarray**‐based transcriptome profiling. One of the pioneering high‐throughput miRNA studies used microarrays to demonstrate that a single miRNA can reduce mRNA levels of hundreds of genes.^146^ The same study also revealed that ectopic expression of miRNA mimics could alter the expression profile of an entire cell. Thus, transfection of the brain‐enriched *miR‐124* changed the profile of HeLa cells toward a neuronal blueprint, while the muscle expressed *miR‐1* induced a muscle‐like profile suggesting that miRNAs participate in establishing tissue specific gene expression.^146^ Microarrays continue to be widely used for transcriptome profiling especially in cancer research.^147^, ^148^ However, the advent of high‐throughput RNA sequencing (**RNAseq**) approaches (Figure 2(a)), established an entirely new standard of sensitivity and coverage in genome‐wide profiling studies. By comparing published *miR‐155* microarray data with RNAseq profiling of *miR‐155* transfected cells, Xu and colleagues demonstrated that RNAseq can identify a substantially larger miRNA targetome than microarrays.^149^ In general, RNAseq is more accurate and able to detect a wider range of expression levels, which are crucial considerations for miRNA research, since miRNAs tend to have relatively mild effects on most of their targets. Moreover, RNAseq can distinguish all gene isoforms and transcripts that differ only by the length of their 3′UTRs. Furthermore, in combination with other techniques (see other sections) RNAseq provides an opportunity for single‐nucleotide resolution analysis and enables exact mapping of RBP motifs. Therefore, based on its intrinsic advantages over microarrays, refined features, and continuous platform evolution, RNAseq should be considered the gold standard for profiling studies in miRNA research today.

This notion is further supported by the recent development of a powerful computational approach for analysis of RNAseq data termed exon‐intron split analysis (**EISA**).^150^ In essence, this study proposed that by comparing intronic read counts, which are usually discarded during data analysis, with variations in exonic read counts, both transcriptional and post‐transcriptional changes in gene expression can be inferred from standard RNAseq experiments (Figure 2(a)). Extrapolating this ingenious conceptual framework to miRNA studies could provide a powerful strategy for discriminating primary versus secondary miRNA targets by simple RNAseq profiling following perturbation of miRNA homeostasis. Indeed, RNAseq profiling at two time points (12 and 32 h) in HeLa cells overexpressing *miR‐1*, revealed that at 12 h a decrease in read counts was only observed in exons but not in introns, suggesting direct *miR‐1*‐mediated post‐transcriptional repression of these genes. However, at 32 h perturbations in both intronic and exonic read counts were detected for several other genes. Supporting the assumption that these reflect secondary effects, bioinformatics target prediction revealed that these genes are devoid of *miR‐1* predicted MREs in their 3′UTRs. This analysis was extended to other miRNAs and cell types with similar outcomes.^150^ Although EISA results can be influenced by a number of secondary factors and should be cautiously interpreted, this platform undoubtedly adds an additional layer of interpretability and resolution to RNAseq profiling in miRNA studies, without increasing overall costs or experimental burden.

### Proteome Profiling

A traditional, but increasingly less utilized, strategy to quantify expression changes at the whole proteome level is *two‐dimensional differential gel electrophoresis* (**2D‐DIGE**), which compares two fluorescently labeled proteomes first by isoelectric focusing and then by molecular weight. Areas of the gel that exhibit differences in fluorescence levels are excised and analyzed by mass spectrometry. This approach has been used to identify targets of *miR‐21* 151, 152 and *miR‐210* 153 in cells in culture following miRNA inhibition or overexpression, and is also applicable to tissue samples as reflected by the identification of miRNA–target pairs in rat kidneys.^154^

More advanced approaches use specific peptide labeling techniques to analyze in parallel, in a multiplex fashion the entire proteome from various samples by mass spectrometry. A popular chemical method for labeling peptides for relative and absolute quantification is the use of isobaric (same mass) tags, which are covalently linked to the N‐terminus of peptides and amines of side chains (**iTRAQ**). The mass difference between experimental and control sample is achieved by releasing a reporter ion that is indicative of each specific label during MS/MS fragmentation.^155^ This method was used to identify potential targets of *miR‐21* in MCF‐7 breast cancer cells.^156^ iTRAQ has also been applied to the analysis of miRNA–target interactions in tissue samples from patients. For instance, this approach was used to highlight a potential role for the *miR‐320a‐Arf1* axis in patients suffering from osteopetrosis,^157^ and to implicate *miR‐128* in regulating prostate cancer invasion.^158^

Another frequently used chemical protein tag is the *isotope‐coded affinity tag* (**ICAT**), which uses biotinylated labels that react solely with cysteine side chains. This exclusive specificity however, is at the same time a limitation of this technique since it can only quantify cysteine‐containing proteins. The labels contain either normal or heavy isotopes (usually carbon or hydrogen), which are used to tag both the experimental and control samples.^155^ After mixing the samples, proteins are digested and the biotinylated end of the label is used to affinity purify tagged peptides on a streptavidin column. Peptides are eluted from the column and the biotin tag is cleaved off prior to mass spectrometry. ICAT has been used to identify targets of *miR‐34a* in IMR32 cells.^159^ In this case, 1495 proteins could be quantified of which 143 were significantly upregulated and 192 were downregulated following synthetic *miR‐34a* delivery. By comparing the proteomics data to microarray mRNA expression profiling, it was proposed that, within this context, *miR‐34a* represses most of its targets predominantly at the translational level.^159^

*Selected reaction monitoring* (**SRM**) is a targeted approach that does not record the entire mass spectra but focuses on a predefined set of peptides, which are monitored over time during their fragmentation. This substantially increases the sensitivity of detection and allows for quantification of low abundance peptides.^160^ A combination of ICAT and SRM has been used to efficiently screen a large number of potential miRNA targets obtained from *in silico* predictions.^161^ Briefly, by monitoring changes in expression of 161 putative *let‐7* targets between wild‐type and *let‐7* mutants in *C. elegans*, 19 proteins were identified to be upregulated and 10 appeared downregulated. Some of these, such as the zinc‐finger protein ZTF‐7, were further validated as true *bona fide let‐7* targets by complementary genetic rescue experiments, sensor assays, and polysome profiling.^161^ The same approach was applied to 118 predicted targets of the *miR‐58* family. Interestingly, of the 27 proteins that could be quantified, all 18 targets predicted to be shared by all family members were upregulated following *miR‐58* loss of function (LOF), despite the presence of the other seed‐related miRNAs.^161^ A more advanced version of this technology was subsequently implemented by integrating SRM, RNA immunoprecipitation, and microarrays (**RIP‐chip‐SRM**) in *C. elegans*.^162^ In this instance, wild‐type and *miR‐58* mutant worms were used for ALG‐1‐mediated RNA immunoprecipitation (RIP) to obtain a set of high‐confidence targets. These were further analyzed by SRM, and simultaneously total mRNA expression levels were quantified by microarrays. Following integration of both datasets, it was proposed that *miR‐58* might also act predominantly through translational repression.^162^

Proteins can also be labeled in living cells through metabolic incorporation of isotopes. *Stable isotope labeling by amino acids in cell culture* (**SILAC**) makes use of the cell's inability to synthesize particular amino acids, which can be supplied as isotope‐labeled nonradioactive ‘heavy’ versions (usually arginine or lysine) in the culture medium (Figure 2(b)). Control cells are grown in normal medium and, since all proteins are labeled in the experimental condition, both samples can be pooled after cell lysis and processed together for quantitative mass spectrometry.^163^ *Pulsed SILAC* (**pSILAC**) was developed to measure changes in protein production between two samples within a defined time frame. In this instance, both samples are treated with isotope‐labeled amino acids, one with a medium‐heavy and the other with a heavy version, and only newly synthesized proteins containing the isotopes are analyzed^164^ (Figure 2(b)). Both SILAC and pSILAC have been used to investigate the effect of miRNAs on protein levels in various human cancer cell lines (HeLa, HEK293T, MiaPaCa2, WM239A, U266, MCF‐7, and various colorectal cancer cells), as well as in mouse neutrophils by comparing changes in mRNA levels to changes in protein abundance.^56^, ^120^, ^164^, ^165^, ^166^, ^167^, ^168^, ^169^, ^170^, ^171^ Although these studies confirmed that manipulating a single miRNA could affect hundreds of proteins, it was concluded that the effect on proteins is often mild (rarely above a 4 fold change) and the strongest repression usually correlates with the presence of 7mer or 8mer sites in 3′UTRs. Overall, target genes exhibiting robust repression of protein levels also displayed a coordinated decrease in mRNA abundance, whereas the consequence of translational interference alone appeared to correlate with only modest degrees of repression. However, another study proposed that for certain miRNAs translational repression might play a more dominant role in miRNA‐mediated regulation.^169^ Finally, pSILAC proteome‐wide analysis of *miR‐22* repression revealed a bimodal threshold of regulation relative to exogenous miRNA levels, with the strongest effect on targets at low and high miRNA concentrations.^120^ A limitation of SILAC approaches is that they are only applicable to cells that are dependent on essential amino acids and can be cultured for at least a few cycles of replication to allow incorporation of labeled amino acids.

Unlike SILAC, ICAT or iTRAQ, **label‐free proteomics** analyses peptides from samples without addition of tags prior to mass spectrometry. Although a cost‐effective alternative to other techniques, this method is not immediately amenable to parallel sample quantification. However, this strategy was employed to assess the effect of *miR‐7* overexpression on protein levels in CHO cells, which are frequently used for the industrial production of recombinant proteins. Interestingly, a dominant proportion of downregulated genes encoded for ribosomal and histone proteins, which could explain the inhibitory effect of *miR‐7* overexpression on CHO cells growth.^172^ A recent study demonstrated that this method could be successfully applied to large‐scale analysis of formalin fixed paraffin embedded (FFPE) tissue samples originating from 106 breast cancer patients. In this study, 100 proteins and 19 miRNAs appeared to be differentially expressed between estrogen receptor positive (ER+) and triple‐negative breast cancer patients, confirming their distinct metabolic profile.^173^

Integrated approaches comparing transcriptomic and proteomic data have been relatively frequently employed to distinguish miRNA targets that are repressed at the translational level from those that are regulated through mRNA destabilization. However, proteomics approaches are in general less sensitive (detection ranges between ~500 and 6000 proteins) and biased toward highly abundant and soluble proteins. Therefore, the correlation of RNA expression data (RNAseq or microarrays) to protein expression data (mass spectrometry) is not always trivial, and the dependability of these approaches is to some extent questionable. Consequently, more sophisticated and reliable technologies have been developed, which endeavored to sample at high resolution the functional consequence of miRNA binding to cellular mRNA targets.

### Translational Profiling

One advanced method that was originally employed to differentiate the impact of miRNAs on translation versus mRNA stability is ***polysome profiling***. This technique measures the number of mRNAs bound by at least one ribosome (ribosome occupancy) as well as the average number of ribosomes per 100 bp (ribosome density). In this assay, cells are treated with cycloheximide to arrest translating ribosomes prior to cell lysis. Ribosome‐bound mRNAs are then separated from the unbound fraction by sucrose gradient ultracentrifugation and quantified by microarrays. This method was used to measure the effect of *miR‐124* overexpression in HEK293T cells, which revealed that about 75% of the targets were repressed at the mRNA level.^58^

***Ribosome profiling*** is an advanced version of polysome profiling in which the microarray readout is replaced by RNAseq, an implementation that permits nucleotide resolution quantification in the binding data. This technique provides a semi‐quantitative measure for translation efficiency and was adopted for miRNA research as an alternative to proteomic approaches to assess the effect of miRNAs on protein production. In this instance, following treatment with cycloheximide the RNA is purified from cell extracts and digested with RNase I to obtain fragments of single ribosomes (monosomes) bound to RNA. A proteinase K treatment step releases the bound RNA fragments, which can be used for cDNA library preparation followed by deep sequencing (Figure 2(c)). Ribosome profiling enables quantification of the number of ribosomes bound to a single mRNA as well as establishing the exact position of each bound ribosome at sub‐codon resolution.^174^ These readouts are more directly comparable to RNA quantification than proteomics approaches. A potential limitation of this strategy is the relatively large amount of material required and the necessity of a sucrose gradient ultracentrifugation step, a technique that is not immediately amenable for all laboratories. This minor technical inconvenience however, has already been solved by the implementation of a standardized size‐exclusion chromatography step in recently developed commercial kits such as the ARTseq/TruSeq Ribo Profile from *Illumina*. Ribosome profiling revealed that mRNA destabilization is the predominant mechanism underlying mammalian miRNA‐mediated post‐transcriptional regulation, while translational repression on its own appears to play a relatively minor role.^59^, ^175^ A more recent model of repression dynamics proposed that translational inhibition represents an immediate rapid consequence of miRNA targeting causing weak initial repression, followed by irreversible mRNA destabilization and decay.^175^ Similar results have been previously observed in zebrafish where *miR‐430* inhibits translation initiation prior to mRNA decay,^60^ and in *Drosophila* S2 cells where it was also demonstrated that translational repression occurs prior to deadenylation and mRNA degradation.^61^ Nonetheless, despite substantial efforts to elucidate the relative contribution of translational inhibition and mRNA destabilization to miRNA‐mediated repression as well as the timing of these events, this topic remains controversial.^175^

The main caveat of both transcriptome and proteome profiling‐based technologies lies in their inability to experimentally discriminate between direct and indirect miRNA targets. To obtain more unequivocal evidence of miRNA–target binding interactions, a number of powerful approaches have been developed which leverage the fact that the association between miRNAs and their targets is mediated by the miRISC. Most of these strategies rely on the immunoprecipitation of miRISC components to pull down associated miRNAs and their targets, which are subsequently profiled at a genome‐wide scale by microarrays or RNAseq.

## CAPTURE‐BASED APPROACHES

RBPs facilitate a multitude of biological processes ranging from nascent RNA processing, nuclear export, RNA localization, translational timing and, perhaps most crucially, RNA stability and turnover. Most of the techniques discussed in this section take advantage of the fact that direct binding of proteins to cellular RNAs protects the bound sequence from RNase‐mediated degradation. Sequencing of the protected fragments allows high‐resolution mapping of the binding interface, and this relatively simple principle has been creatively exploited to develop a range of powerful approaches for high‐throughput identification of miRNA–target binding events.

### RIP, RIP‐Chip, and RIP‐Seq

The most straightforward and reliable methods to identify miRNA–target interactions *in vivo* relied on the immunoprecipitation of either wild‐type or tagged versions of Ago, which directly contacts both the miRNA and their mRNA targets. Ago‐bound co‐immunoprecipitated RNAs are extracted and either analyzed by qRT‐PCR, microarray (RIP‐Chip), or next‐generation sequencing (RIP‐seq).^58^, ^153^, ^162^, ^176^, ^177^, ^178^, ^179^, ^180^, ^181^, ^182^, ^183^ Other components of the miRISC protein assembly however, have also been exploited for this purpose. For example, GFP tagged versions of the two *C. elegans* GW182 orthologs, AIN‐1 and AIN‐2, have been used to identify miRNA–target binding events either in whole animals,^184^, ^185^ or in specific tissues by targeted expression of the fusion protein.^186^ A clever adaptation of this approach termed ***RISCtrap*** used a dominant negative form of the human paralog of GW182 (hTNRC6A) that can still bind Ago but inhibits silencing and degradation of the bound mRNA targets, which remain trapped within the miRISC.^187^ This approach allows simultaneous analysis in a single experiment of miRNA‐targets bound by several different Ago proteins. *RISCtrap* was used to identify targets of *miR‐124*, *miR‐132*, and *miR‐181* in HEK293T cells. Interestingly, although most *miR‐124* and *miR‐132* MREs appeared to be located in the 3′UTR of genes, *miR‐181* C2H2 class zinc‐finger targets contained MREs enriched in the C2H2 motif repeats located within the ORF. The majority (>60%) of the identified targets contained canonical 8mer, 7mer‐m8, or 7mer‐1A sites.

Although useful, RIP approaches bear the risk of high false positive discovery rates as a consequence of low stringency purification protocols necessary to preserve protein–RNA interactions (reviewed in Ref 188). This inherent limitation results in unspecific co‐immunoprecipitation of RNA species from a cellular extract, due to the propensity of proteins and RNAs to ‘stick’ to each other *via* nonspecific electrostatic interactions. This has direct relevance to miRNA research since it has been reported that cell lysis can promote re‐association of RBPs with RNAs and thus may result in artificial miRNA‐Ago interactions.^189^, ^190^ Therefore, to reduce the risk of unspecific binding of RNA to proteins during the IP step, stabilization of physiological *in vivo* interactions allowing high stringency purification conditions became mandatory.

### CLIP and CLIP Variants

**Figure 3:**
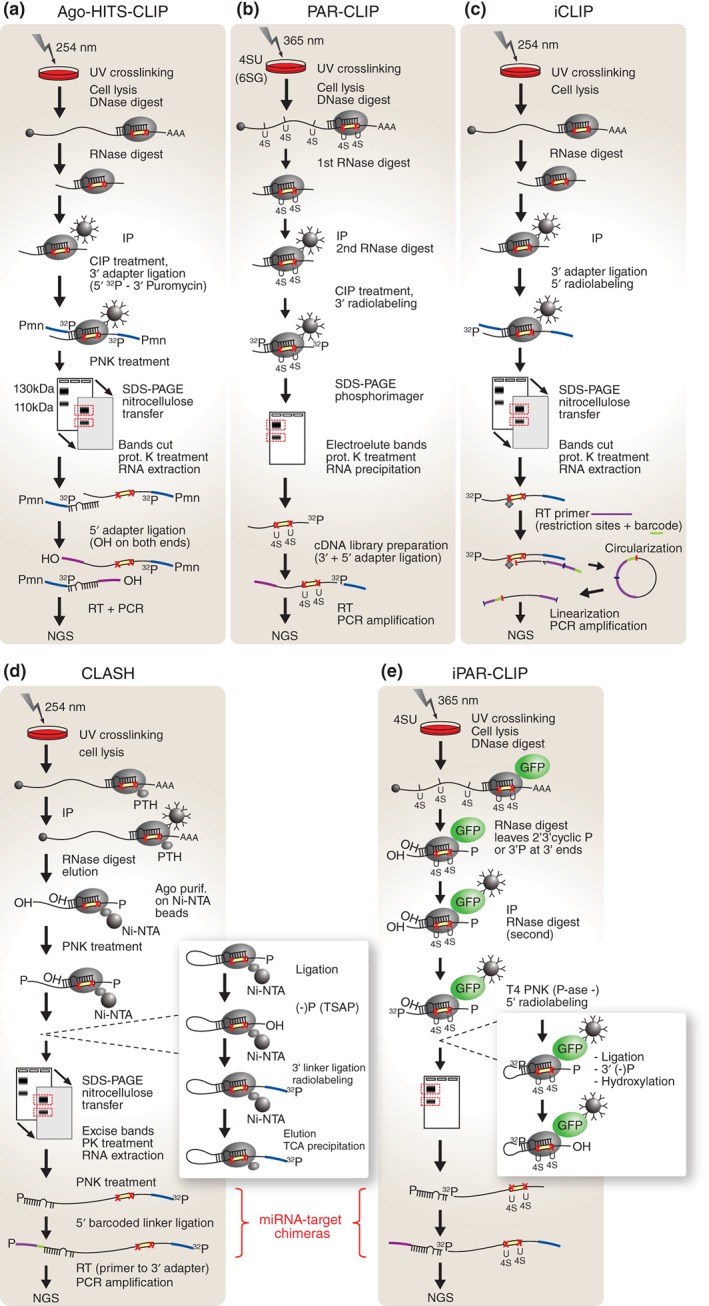
CLIP‐based high‐throughput miRNA target identification strategies. Specificity is achieved in all CLIP technologies by UV crosslinking (red X denote crosslinking sites) and size selection of Ago‐mRNA‐miRNA ternary complexes (SDS‐PAGE). All approaches use high‐throughput sequencing to quantify the purified RNA. (a) HITS‐CLIP yields two separate sets of data, one for mRNAs and one for miRNAs. (b) In PAR‐CLIP 4SU or 6SG is incorporated into RNA to enhance crosslinking and pull‐down efficiency. Only the coprecipitated mRNA is used to map the Ago‐binding sites with high resolution. (c) In iCLIP a special barcoded primer allows for the recovery of RT fragments that have been terminated at crosslinking sites due to protein remnants (diamond close to red X) resulting from incomplete crosslink reversal. The primer allows for circularization of fragments and subsequent linearization, *via* an internal restriction site, generating adapters on both ends of the fragments. These fragments are used to map Ago‐binding sites with high resolution. (d) The CLASH protocol introduces an additional Ago‐mRNA‐miRNA purification step on Ni‐NTA beads and ligates the 3′ end of the miRNA to the 5′ end of target mRNA to obtain miRNA–target chimeras. (e) In iPAR‐CLIP 4SU is used to increase crosslinking efficiency and an Ago‐GFP fusion protein for the IP. This method also includes a ligation step to link the 3′ end of the miRNA to the 5′ end of the target mRNA generating miRNA–target chimeras. UV, ultraviolet light; 4SU, 4‐thiouridine; 6SG, 6‐thioguanosine; NGS, next‐generation sequencing; RT, reverse transcription; CIP, calf intestinal alkaline phosphatase; IP, immunoprecipitation; Ni‐NTA, nickel‐charged affinity resin (nitrilotriacetic acid); TCA, trichloroacetic acid; PTH, protein A + TEV protease cleavage site + 6xHis tag; P, phosphate; OH, hydroxyl; PNK, polynucleotide kinase; T4 PNK (P‐ase ‐), T4 polynucleotide kinase (3′ phosphatase minus); prot. K, proteinase K; TSAP, thermosensitive alkaline phosphatase; GFP, green fluorescence protein; SDS‐PAGE, sodium dodecyl sulfate polyacrylamide gel electrophoresis; Pmn, puromycin.

The first crosslinking‐based high‐throughput approach applied to miRNA research, was *Argonaute high‐throughput sequencing of RNAs isolated by crosslinking and immunoprecipitation* (**Ago‐HITS‐CLIP**)^83^, ^191^ (Figure 3(a)). HITS‐CLIP was originally developed as a method to map RBPs to mRNAs^192^ and has been recently refined to allow single‐nucleotide resolution analysis by integration of *cross‐link‐induced mutation site* (CIMS) maps.^193^, ^194^ Briefly, this method uses UV irradiation to covalently crosslink miRNAs and their target mRNAs to Ago proteins in cells or tissue. After cell lysis, RNA fragments are trimmed by RNase treatment and complexes are subsequently immunoprecipitated with an Ago antibody. miRNA‐mRNA‐Ago ternary complexes are then separated from miRNA‐Ago binary complexes by radiolabeling of RNA followed by SDS gel purification and nitrocellulose membrane transfer. Following crosslink reversal and removal of Ago by proteinase K treatment, RNA is isolated from the membrane and reverse transcribed into a cDNA library, which is then subjected to high‐throughput sequencing (Figure 3(a)). Since miRNA‐mRNA complexes are dissociated prior to sequencing, two separate data sets are obtained, one for the miRNAs and one for their targets. These are subsequently correlated by matching the Ago footprint to putative miRNA target sites, and ternary binding maps are generated using an established bioinformatics pipeline for data analysis.

Ago‐HITS‐CLIP has been successfully applied to the analysis of miRNA targetomes in the mouse brain,^83^ many different human and mouse derived cell lines,^21^, ^83^, ^195^ as well as *C. elegans* larvae at developmental stage L4.^196^ This method was also used to identify targets of viral miRNAs in KSHV‐infected or in EBV‐infected B cells.^197^, ^198^ These studies confirmed previous findings that miRNA binding sites are not only restricted to the 3′UTR of genes but can also occur in the 5′UTRs and coding DNA sequence (CDS). However, the relevance of these sites from a regulatory capacity perspective remains controversial. For example, in mouse embryonic stem cells miRNA binding sites in the CDS appear to regulate mRNA levels with a similar efficacy to MREs in the 3′UTR.^195^ However, in ALG‐1 mutant *C. elegans* no upregulation of mRNA levels was observed for genes with miRNA binding sites mapping to CDS regions.^196^ A recent analysis of several datasets obtained through various methods suggested that sites in the CDS preferentially mediate translational repression, while miRNA binding to 3′UTRs results predominantly in mRNA destabilization.^199^

A surprising discovery which emerged from HITS‐CLIP‐based studies was that miRNA binding to many cellular targets can also be mediated *via* imperfect noncanonical interactions. Understandably, the first bioinformatics pipelines for generating genome‐wide miRNA–target binding maps from Ago‐HITS‐CLIP data only took into consideration canonical perfect seed‐matched MREs. Surprisingly however, this initial analysis revealed a large number of so called ‘orphan clusters’, which comprised of all Ago‐crosslinked mRNA tags that did not map to canonical MREs as defined by perfect complementarity across the seed region of the miRNA.^83^ A detailed motif analysis of *miR‐124* orphan clusters revealed that in fact many contained a specific mismatch to the seed sequence of the miRNA, in the form of a G insertion at position 6. It was proposed that consecutive binding at nucleotides 2–6 of the miRNA introduces a G‐bulge in the MRE creating a functional target site, which can be bound and regulated by *miR‐124*.^23^ Based on its function in this alternative miRNA–target interaction mechanism, position 6 was termed the ‘*pivot*’ nucleotide. Subsequent analysis revealed that bulge nucleation sites are present in the binding motifs of other miRNAs and appear to represent an evolutionary conserved phenomenon.^23^ A different type of noncanonical MRE discovered in around 40% of *miR‐155* target sites, contained a single mismatch in the seed. However, such noncanonical sites were found to be less repressed than perfect seed‐matched MREs^21^ or nonconsequential.^29^ Another frequently occurring alternative MRE type was observed in *C. elegans*, and it was defined by a perfect seed with a single G:U wobble base‐pair.^196^ Interestingly, a HITS‐CLIP study in mouse embryonic stem cells identified a G‐rich motif that appeared to be bound by Ago2 in both a miRNA‐dependent and independent manner.^195^ Although this interaction did not seem to confer repressive activity in the absence of miRNAs, it was proposed that it may increase the affinity of Ago2‐miRNA binding to targets when present in close proximity of an MRE, thus augmenting miRNA‐mediated regulation. However, it remains unclear whether Ago2 itself binds to these G‐rich motifs or if other proteins of the miRISC facilitate this interaction.^195^ In *C. elegans* ALG‐1 binding sites appeared to also be present in CDS and 5′UTRs in addition to 3′UTRs of genes. However, in this instance the sites located in 3′UTRs appear to carry a distinct signature characterized by flanking CU‐rich motifs and greater accessibility, which correlated with increased activity.^196^

To improve crosslinking efficiency and enable generation of high‐resolution interaction maps for RBP and miRISC binding sites across the entire transcriptome, a variation of HITS‐CLIP termed *Photoactivatable Ribonucleoside Enhanced Crosslinking and Immunoprecipitation* (**PAR‐CLIP**) was developed^200^, ^201^, ^202^, ^203^ (Figure 3(b)). The distinctive feature in the PAR‐CLIP protocol is the addition of 4‐thiouridine (4SU) or 6‐thioguanosine (6SG) photoactivatable nucleosides to the cell culture media, which can be randomly incorporated into nascent transcripts. These nucleosides are crosslinked with increased efficiency to bound proteins by UV irradiation at 365 nm (Figure 3(b)). Furthermore, incorporation of 4SU induces the transition of thymidine to cytidine in cDNA libraries. Because this conversion occurs with significantly higher frequency at crosslinked sites than the rest of the genome, it allows high‐resolution mapping of RBP motifs. This phenomenon was exploited to identify miRNA‐mRNA binding events using tagged Ago immunoprecipitation in HEK293 cells.^200^ This study further confirmed that miRNA binding sites are not restricted to 3′UTRs but are also frequently found (50%) in the CDS and 5′UTR of genes. However, sites in the CDS and 5′UTR appeared to only marginally cause mRNA destabilization compared to those encoded in 3′UTRs, despite their striking similarity in sequence and structure. In addition, PAR‐CLIP has been used to identify the targetome of viral miRNAs in KSHV‐infected primary effusion lymphoma (PEL) cells,^204^ EBV‐infected lymphoblastoid cell lines (LCLs),^205^ and MCF7 breast cancer cells.^206^ This approach also confirmed the existence of noncanonical MREs but at a lower rate. Only about 6% of the bound target sites were found to contain G:U and U:U wobbles, or mismatches in their seed complementary sequences. Interestingly, PAR‐CLIP studies revealed that auxiliary sites outside the seed region (positions 13–15) can weakly support miRNA–target interactions under certain circumstances.^200^

Although PAR‐CLIP provides a strategy to filter true signal from noise by detection of miRNA binding sites based on T > C positional mutation frequency, it is not suitable for the analysis of most tissue or *in vivo* studies, were photoreactive nucleosides cannot be easily delivered to cells prior to crosslinking. Furthermore, it has been shown that 4SU can be toxic to cells and alter their physiological homeostasis.^207^, ^208^ Lastly, from a technical standpoint, incomplete crosslink reversal by proteinase K treatment is an important consideration in PAR‐CLIP studies. This can result in persistent ‘peptide stubs’ associated with the RNA which can lead to termination of reverse transcription (RT) reactions at the crosslinking site, a phenomenon that was also observed in HITS‐CLIP data.^194^, ^209^

To alleviate some of these limitations, an improved method called *individual‐nucleotide resolution CLIP* (**iCLIP**)^210^, ^211^, ^212^ was recently adapted to miRNA research^213^ (Figure 3(c)). Briefly, iCLIP relies on the ligation of a special adapter promoting circularization and subsequent linearization by restriction endonuclease digestion, to isolate all RT cDNA products including those originating from truncated transcripts at crosslinked sites. In addition, iCLIP uses barcoded primers for cDNA library preparation to eliminate PCR artifacts (Figure 3(c)). iCLIP was successfully employed to identify with high sensitivity and improved resolution Ago‐binding sites in *C. elegans*.^213^ This method has also been recently used to analyze the susceptibility of miRNAs to competitive endogenous target inhibition in mouse embryonic and mesenchymal stem cells.^214^ Based on this study, it was estimated that miRNAs with low miRNA:target ratios (*miR‐92/25*) would require approximately 3,000 additional high‐affinity binding sites (7mer or 8mer) to outcompete their pool of endogenous targets. However, for highly abundant miRNAs such as *miR‐294* and *let‐7*, even 10,000 copies of a competing endogenous RNA (ceRNA; see ‘Conclusions and Outlook’ section) would not be sufficient to impact their activity and attain a comparable effect.

While PAR‐CLIP and iCLIP aimed to enhance the sensitivity and resolution of RBP interaction maps, a new method termed *crosslinking, ligation, and sequencing of hybrids* (**CLASH**)^215^ set out to improve identification of RNA–RNA interactions by generation of intermolecular hybrids (Figure 3(d)). This method was also adapted to miRNA research by including an RNA ligation step in the Ago‐HITS‐CLIP protocol which covalently links miRNAs to their bound target mRNAs, followed by high‐throughput detection of miRNA–target chimeric reads by next‐generation sequencing^20^ (Figure 3(d)). Intriguingly, although the coverage depth was low with only 2% of the sequencing reads representing actual hybrids, this study suggested that noncanonical seed pairing (G:U pairs, or a single mismatch) were approximately 1.7 fold more frequent than canonical seeds with perfect base pairing. Furthermore, the analysis of binding patterns among all detected hybrids, revealed the presence of five distinct MRE classes. These classes displayed a wide range of interactions such as strict 5′ seed pairing, involvement of the miRNA 3′ end, exclusive binding outside of the seed sequence (nearly 18% of hybrids), and diffuse binding (see Figure 1). A similar approach was used in *C. elegans* by incorporating a ligation step in the PAR‐CLIP protocol (**iPAR‐CLIP**) to enable unambiguous detection of miRNA–target binding events^22^ (Figure 3(e)). This integrated approach allowed generation of both high‐resolution Ago‐binding maps based on T > C mutation frequency (PAR‐CLIP) and thousands of chimeric reads (CLASH), most of which mapped to 3′UTRs and almost all could be assigned to Ago‐binding sites. Analysis of the miRNA–target binding patterns revealed that approximately 80% were complementary to the miRNA (seed) with up to two mismatches or bulges. In general, mismatches occurred most frequently at position 2 and 7 of the seed. Surprisingly, chimeras were also detected in control samples devoid of recombinant ligase suggesting that endogenous ligases present in cell lysates can also facilitate hybrid RNA formation after RNase treatment. Since all CLIP approaches used RNase‐mediated trimming of Ago‐bound RNAs, under this premise chimeric reads should have been generated in all previous studies but went undetected during data analysis. Indeed, revisiting seven previously published Ago‐HITS‐CLIP datasets confirmed the presence of miRNA–RNA chimeras, and the ensuing binding patterns displayed a similar enrichment for seed interactions with up to two mismatches.^22^

Perhaps one of the most challenging aspects of Ago‐CLIP‐based technologies is the complex bioinformatics analysis required to demultiplex sequencing reads and assemble them into miRNA–target binding maps. Consequently, several algorithms, and automated platforms have been developed to facilitate this process in CLIPseq studies. For example, **MIRZA** is a biophysical model for calculating miRNA–target binding interactions which is able to predict canonical as well as noncanonical MREs based on energy parameters inferred from CLIP data.^27^ Similarly, **microMUMMIE** 28 and **PARma** 216 identify miRNAs emerging from PAR‐CLIP binding data. Other algorithms were designed as stand‐alone all‐inclusive platforms available online, which are capable of performing a complete analysis of CLIP data. These tools include **PARalyzer** for PAR‐CLIP datasets,^217^ **miRTarCLIP** for HITS‐CLIP and PAR‐CLIP experiments,^218^ and **PIPE‐CLIP** for HITS‐CLIP, PAR‐CLIP, and iCLIP data.^219^ Since most studies compare different experimental conditions, an algorithm called **dCLIP** was specifically designed for quantitative analysis across various CLIPseq experiments.^220^ Additionally, several databases and analysis environments have been created to integrate and annotate published CLIPseq data, including **CLIPZ**,^221^ **starBase** 222 and **starBase v2.0**,^223^ **doRiNA**,^93^ and **TarBase 6.0** 224 which has recently been updated to **DIANA‐TarBase v7.0.** 225

CLIP approaches are undoubtedly the current state‐of‐the‐art technologies for mapping miRNA–target binding events, and are generating reproducible and robust data which allows analysis across different strategies as well as various biological contexts. This was elegantly demonstrated by a comparison of HITS‐CLIP and PAR‐CLIP data, which showed that both techniques produced very similar results.^221^, ^226^ A cross‐analysis study over 34 published Ago‐CLIPseq datasets obtained from different mammalian cell lines and tissues revealed that in all cases MREs are distributed across 3′UTRs, 5′UTRs, and CDS, albeit with different frequencies.^226^ In the same study, an analysis of miRNA seed architecture across all datasets revealed that only 3–12% of the MREs accounted for perfect complementary seed sites. The most frequently found nonperfect interactions were seed with G:U wobbles in combination with a bulge in the miRNA (30–50%). Slightly less frequent were G:U wobbles in the seed paired with a bulge in the target (20–40%), or without any bulge (15–25%).^226^

### RNA‐Bait Approaches

An alternative approach for interrogating miRNA–target interactions employs mRNAs as ‘baits’ to pull down associated miRNAs, or conversely, modified miRNAs to capture their potential mRNA targets. A routinely used system for mRNA tagging and purification is the bacteriophage‐derived MS2 stem‐loop sequence, which is specifically recognized by an RBP called the MS2 coat protein (MCP). By fusing MCP to various affinity tags, small RNA species can be copurified with a candidate MS2‐containing mRNA (Figure 2(d)). This method was used to discover miRNAs regulating *Hand2* in primary rat cardiomyocytes.^227^ In this instance, three MS2‐loops were cloned into the *Hand2* 3′UTR and delivered to cells using a lentiviral expression system. *Hand2* mRNA was then captured from cell lysates by affinity purification using MCP fused to maltose binding protein (MBP) and an amylose column. Copurified miRNAs were subsequently analyzed on an ABI Taqman multiplex microRNA array, which confirmed *Hand2* targeting by *miR‐1* and identified *miR‐133a* as a novel miRNA regulating *Hand2* expression in tissue cultures and mice. A similar approach generically termed **MS2‐TRAP** was used to study miRNAs associated with *lincRNA‐p21* in mouse embryonic fibroblast (MEF) cells.^228^ In this case, 24 MS2 hairpin loops were appended to 3′ end of the lincRNA, and a MCP‐GST fusion protein was used to isolate this transcript from cell lysates. Four of five miRNAs predicted to target *lincRNA‐p21* (*let‐7b*, *let‐7c*, *miR‐130*, *miR‐221*) were found to be enriched in the pull‐down fraction by a targeted poly(A) tailing qRT‐PCR assay.^228^

An *ex vivo* variation of this technology was developed under the name *miRNA trapping by in vitro RNA affinity purification* (**miTRAP**)^229^ (Figure 2(d)). In this case, *in vitro* transcribed 3′UTRs of candidate target genes tagged with four MS2 loops were immobilized on an amylose resin *via* MCP‐MBP fusion proteins, and incubated with cell lysates. Cellular miRNAs binding to bait 3′UTRs under these *ex vivo* conditions could be analyzed either on an individual basis by TaqMan qRT‐PCR or in a high‐throughput fashion by RNAseq. miTRAP was employed to identify miRNAs associating with *ZEB2‐3′UTR* and *MYC‐3′UTR* from U2OS or HEK293 cell lysates. Using *MYC‐3′UTR* as bait, this study identified 18 novel *MYC* miRNAs, of which eight targeted *in silico* predicted MREs while the other ten belonged to miRNAs targeting noncanonical binding sites. Based on these findings, it was proposed that *MYC* might be regulated by multiple miRNAs exerting a combinatorial effect.^229^ In addition to MS2, *in vitro* transcribed RNAs can also be tagged by incorporation of biotinylated nucleotides (Figure 2(d)). This system has been used to immobilize the 3′UTRs of *TCF8* and *Smad4* on streptavidin‐coated magnetic beads.^230^ Following incubation with cellular miRNA pools isolated either from normal human bronchial epithelial (NHBE) cells or mouse liver tissue, captured miRNAs were cloned and sequenced. Several miRNAs were identified which appeared to bind both the *TCF8* and *Smad4* 3′UTRs. Since these *in vitro* tagging approaches do not require expression of ectopic constructs, they are in principle applicable to the analysis of patient samples.^230^

**miR‐CATCH** is a platform enabling isolation of native transcripts using a biotinylated DNA capture oligonucleotide complementary to a mRNA of interest^231^ (Figure 2(e)). Using this protocol, *alpha‐1 antitrypsin* (*AAT*), *interleukin‐8,* and *leucoprotease* inhibitor mRNAs were tested in three different human cell lines (THP‐1, 16HBE14o‐, and HepG2). In this case, copurified miRNAs were subsequently identified by qRT‐PCR and *Nanostring* profiling. Noteworthy, this study suggested that *AAT* appeared to be targeted by a different set of miRNAs in each cell type.^231^ A similar study used a mixture of three different biotinylated antisense DNA oligonucleotides against GFP.^232^ In this instance, the 3′UTRs of *PDCD4* and *Eps8* were cloned downstream of GFP and transfected into HeLa cells. Analysis of copurified miRNAs by qRT‐PCR showed enrichment of *miR‐21*/*miR‐499* and *miR‐124*/*miR‐520b* for *PDCD4* and *Eps8*, respectively.^232^

Other studies reported complementary approaches, which take advantage of biotinylated synthetic miRNAs to isolate potential target mRNAs following coprecipitation and quantification by qRT‐PCR or microarrays. For example, this strategy has been employed for *miR‐34a* in two human cell lines (HCT116 and K562),^233^ *bantam* in *Drosophila* S2 cells,^234^ and *miR‐122* in HepG2 and HeLa cells.^235^ Alternatively, *labeled miRNA pull‐down* (**LAMP**) uses digoxigenin (DIG) to tag pre‐miRNAs, which are then incubated with cell extracts. In this case, processing by Dicer to mature miRNAs and target association, are carried out *ex vivo*. Mature miRNAs are captured on anti‐DIG beads and bound target mRNAs are detected by qRT‐PCR or microarrays. This approach was used to identify *Hand2* as a target of zebrafish *miR‐1*.^236^

A more advanced version of these protocols called *tandem affinity purification of miRNA target mRNAs* (**TAP‐Tar**) employs a sequential pull‐down strategy consisting of an initial affinity purification of FLAG‐tagged Ago1 or Ago2 complexes, followed by capture of biotinylated miRNAs on streptavidin coated beads.^237^ This strategy has been originally tested in HeLa cells stably expressing a tagged Ago protein, transfected with a biotinylated synthetic *miR‐20a*. Subsequent qRT‐PCR quantification of the pull‐down RNA species revealed a specific enrichment in the *miR‐20a* putative target *E2F1*.^237^

An alternative approach called **miR‐TRAP**, omits antibody‐mediated Ago purification and relies on streptavidin pulldown of 3′ biotinylated miRNAs that have been fused with photoreactive psoralen (Pso) groups at position 9 from the 5′ end.^238^ Pso is a photoinducible (at 360 nm) RNA‐RNA crosslinker that forms a covalent bond with uridines in target mRNAs. This bond is photoreversible at 254 nm irradiation. When this method was used to isolate the targetome of biotinylated Pso‐miR‐29a in MEF cells, two miR‐29a predicted targets were found to be enriched.^238^ **miR‐CLIP** re‐introduces the immunoprecipitation step, and takes advantage of miRNAs with optimized configurations for coupling biotin and Pso groups (Figure 2(f)). This sequential pull‐down approach has been employed to capture the targetome of *miR‐106a* in HeLa cells, and uncovered approximately 600 enriched targets of this miRNA including the long noncoding RNA *lincRNA H19*.^239^

Although capture‐based approaches have significantly improved our understanding of miRNA–target interactions, they have several important limitations which warrant careful consideration. For example, most RNA‐bait protocols are performed *ex vivo* or at nonphysiological miRNA–target stoichiometry, and therefore the biological relevance of the discovered miRNA–target interactions is uncertain and requires extensive *in vivo* validation. More generally, achieving reliable results with most capture‐based approaches requires large amounts of starting material, which limits these techniques to cultured cells or tissue samples. Therefore, their applicability to single cell experiments or small number of cells isolated by FACS remains a distant and challenging goal. However, it is this level of sensitivity that would be required to study the role of miRNAs in complex regulatory networks during developmental transitions or under pathological conditions. The necessity of large amounts of starting material also carries the inherent risk of a bias toward highly abundant targets. Finally, it is still unclear whether the physical association of a miRNA to mRNAs inevitably translates into productive repression with functional consequences. miRNAs may only bind transiently to MREs without exerting any repressive effect, but sufficient to be captured by a crosslinking snapshot.^16^ In addition, the analysis of CLIP and iCLIP data suggested that crosslinking itself displays a certain bias toward U bases, which may result in preferential detection of some MREs but not others.^240^

A conceptually interesting idea to detect *in vivo* miRNA–target binding events was to utilize miRNAs associated with target mRNAs as primers for RT. This strategy relies on the assumption that since the 3′ end of the miRNA is sometimes base pairing with the target, it should be at least temporarily available for the initiation of an RT reaction. However, this strategy depends on reaction conditions which preserve *in vivo* formed miRNA‐mRNA duplexes, and implies that no artificial associations of other small RNAs and mRNAs could occur during cell lysis. The first approach exploiting this conceptual framework aimed to identify miRNAs that were associated with unique candidate targets.^241^ In this instance, a short RT reaction on the hTERT‐RPE1 cytoplasmic fraction was used to extend bound miRNAs and generate a DNA‐miRNA hybrid. The resulting chimeras were then purified and used for a second round of RT at a higher temperature to further extend the hybrids. Following 5′ adapter ligation and PCR amplification, chimeric double‐stranded cDNAs were cloned into a vector and sequenced. Based on hybrid analysis, a number of possible miRNA–target interactions were proposed including, *miR‐32*/*miR‐129*:*β‐actin*, *miR‐33*/*miR‐17*/*let‐7a*:*K‐Ras*, and *let‐7a*:*N‐Ras*.^241^ The second study used a nearly identical concept, but aimed to discover new targets of a specific miRNA in *C. elegans* extracts.^242^ In this case, RBPs and components of the miRISC were initially removed by a short treatment with a strong detergent at low temperature. miRNAs were then used to prime a RT reaction under gradually increasing temperature conditions. This step was followed by second strand synthesis, cDNA purification, restriction enzyme digest, and 3′ adapter ligation. Resulting cDNA chimeras were PCR amplified using adapter primers and biotinylated primers complementary to either *let‐7* or *lin‐4*. After purification on an avidin column, PCR fragments were cloned and subjected to sequencing. This method yielded 159 clones corresponding to 73 possible *let‐7* target genes. Forty candidate genes were subsequently tested by RNAi in a *let‐7* mutant rescue assay, resulting in the identification of K10C3.4 as a potential target, further validated by sensor assays.^242^ Although conceptually attractive, a number of limitations restrict the scope and applicability of these RT‐based technologies. These include but are not limited to the degree of target complementarity with the miRNA 3′ end, the accessibility of miRNA 3′ end for reverse transcriptase binding and elongation, and the potential erroneous association of miRNAs or other small RNAs with mRNAs due to low stringency protocols (see also RIP).

Finally, a high‐throughput cell‐based reporter assay (**LightSwitch**) has been reported, which measures the expression changes of artificial luciferase sensors in response to miRNA activity.^243^, ^244^ For miRNA target identification in human cells, a genome‐wide 3′UTR reporter library for use in this assay was made commercially available from SwitchGear Genomics. This strategy was used to screen 139 predicted *miR‐122* targets in HT‐1080 fibrosarcoma cells and yielded 37 repressed candidates.^244^ Similarly, a dual‐luciferase sensor assay was used to identify all miRNA interactions within the 3′UTRs of seven candidate genes.^245^ Screening a human miRNA expression library against this subset of sensors in HEK293T cells uncovered that single mRNAs can be repressed by many different miRNAs.^245^

## ELUCIDATING miRNA BIOLOGICAL FUNCTIONS

Although bioinformatics predictions and high‐throughput target identification are pivotal for miRNA research, they do not provide definitive information regarding the biological function of miRNAs. Even when target maps are being constructed in cell‐based systems, they do not necessarily reflect functional miRNA regulatory interactions at organismal level. For example, analogous miRNA–target axes may differ between cell types, developmental stages, or in response to external stimuli. Therefore, the biological roles of miRNAs can be surprisingly complex, and elucidating them oftentimes require combinatorial approaches for disrupting miRNA activity in time and space, as well as under altered physiological conditions such as injury, stress, and disease. The strategies employed to deconstruct miRNA functions can be broadly grouped in two categories: techniques that aim to alter endogenous miRNA levels or activity, and tools for identifying or disrupting functional miRNA–target interactions *in vivo* (summarized in Table 4).

**Table 4:**
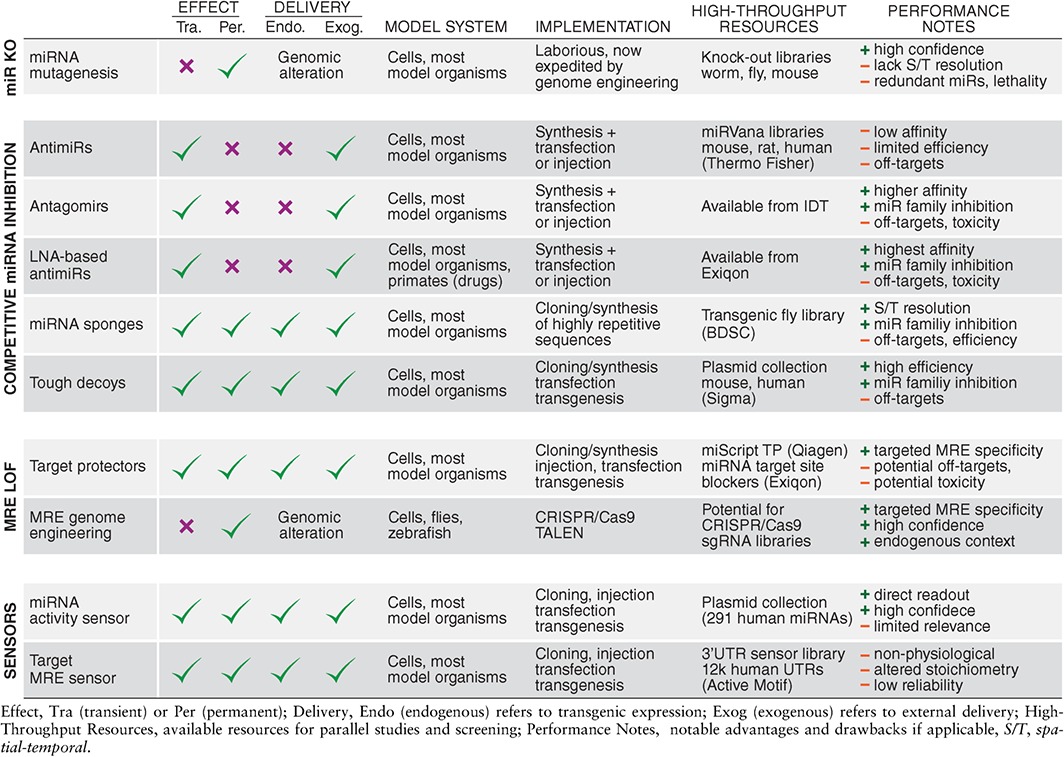
Strategies for Interrogation of miRNA Biological Functions

### miRNA Mutagenesis

**Figure 4:**
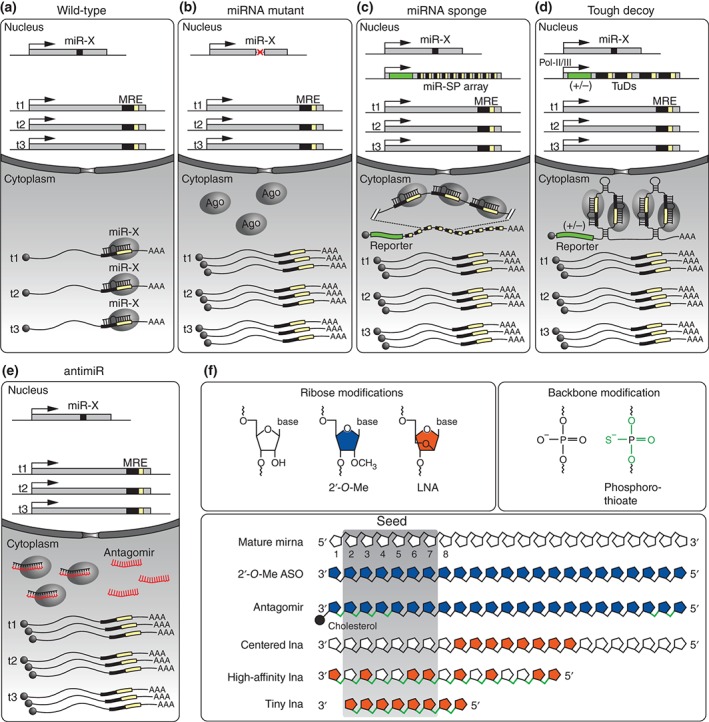
miRNA loss‐of‐function approaches. (a) Under normal homeostasis, endogenous miRNA‐activity results in target repression. (b) Ablation of the genomic miRNA locus enables generation of a null miRNA mutant allele resulting in a global de‐repression of all its targets. (c) miRNA sponge (miR‐SP) competitive inhibitors are genetically encoded into the 3′UTR of a reporter genes and expressed from Pol II promoters. When deployed to *in vivo* they sequester a cognate miRNAs resulting in de‐repression of its endogenous targets. (d) Tough decoys (TuD), are also genetically encoded miRNA competitive inhibitors with enhanced RNA secondary structure for improved targeting and stability. (e) Modified synthetic antimiR oligonucleotides (such as antagomiRs) can be transfected into cells to sequester miRNAs away from their targets. (f) AntimiRs frequently contain chemical modifications which enhance their cellular stability and increase their miRNA binding affinity. Common ribose modifications include 2′‐O‐methyl nucleotides or locked nucleic acid (LNA) incorporations for enhanced affinity. The phosphate backbone can also carry a phosphorothioate modification for increased resistance to RNase degradation. Antagomirs are coupled to cholesterol to aid cellular uptake. LNA nucleotides have been incorporated in the center of long ASOs, spaced throughout the entire length of the ASO. In contrast, tiny LNAs are entirely LNA‐based short 8 bp ASOs complementary to the miRNA seed sequence. MRE, miRNA response element; Pol‐II/III, RNA Polymerase II/III promoters; t1,2,3, miRNA targets; AAA, poly‐A tail; miR‐SP, miRNA sponge; TuD, Tough decoy; ASO, antisense oligonucleotides; yellow box in MRE, seed match region.

The most reliable approach for investigating the functional relevance of a gene is the generation of genomic null mutant alleles for LOF studies (Figure 4(b)). Although a battery of methodologies has been developed in time for the mutagenesis of protein‐coding genes, including but not limited to chemical and physical mutagens (EMS, ENU, UV), synthetic transposable elements, and homologous recombination (HR), their applicability to miRNA studies was challenging. The peculiar nature of miRNAs rendered most of these classic mutagenesis approaches ineffective, or prone to erroneous results. For example, due to their noncoding nature, targeting promiscuity, and very short ‘active’ sequences, single‐nucleotide mutations are in nearly all cases nonconsequential for miRNAs. With regard to genomic architecture, miRNAs are often embedded inside protein‐coding genes or processed from long noncoding transcripts. Consequently, their genomic ablation can result in artefactual LOF or respectively gain of function (GOF) phenotypes associated with the host gene. Therefore, only precise deletions limited to the corresponding mature miRNA sequence or precursor hairpin locus should be considered when attempting to generate null miRNA mutants by genomic ablation. Alternatively, the ideal situation would be to replace the mature miRNA sequence in the precursor locus with a scrambled sequence, thus generating a miRNA null allele without interfering with processing the precursor hairpin from the primary transcript. This however, limits the available mutagenesis methodologies to HR, or the recently developed genome engineering‐based strategies (see below) for homology‐directed repair (HDR). Finally, because miRNAs can belong to functionally redundant ‘seed’ sequence families encoded at multiple distant loci, LOF studies are relevant only when all members of the family have been ablated and genetically crossed into the same animal.^142^, ^246^

Nonetheless, with careful consideration, null miRNA mutants remain the gold standard for LOF studies, and have undoubtedly been at the foundation of most of our current knowledge regarding the roles of miRNAs in development and disease. Indeed, a multitude of targeted or accidental mutagenesis experiments account for the discovery of many fascinating miRNA biological functions. For example, the first miRNA target axis, *lin‐4*:*lin‐14*, was discovered in *C. elegans* in 1993.^3^, ^4^ However, both genes have been identified more than a decade earlier as mutants that could either lead to precocious execution of late larval developmental programs (*lin‐14*) or repeatedly initiate developmental events typical for the first larval stage (*lin‐4*).^247^, ^248^, ^249^ Owing to the epistatic nature of the two mutants it became clear that *lin‐4* had to be a negative regulator of LIN‐14 protein which eventually led to the discovery of the first miRNA.^3^, ^4^

Perhaps one of the most defining studies in miRNA research was the discovery 7 years later of the *let‐7* heterochronic miRNA in a genetic screen for mutations suppressing synthetic lethality.^250^ The implication of *let‐7* in controlling developmental timing events mediated by *lin‐14* repression, and more importantly its conservation from worms to humans, opened the flood gates of miRNA research.^251^ Since then, many landmark studies in various model organisms have relied on genetic manipulation of miRNA loci to uncover widespread functions of miRNAs in a variety of biological processes, including cell proliferation, differentiation, survival, apoptosis, stem cell maintenance, lifespan and aging.^7^, ^8^, ^252^, ^253^, ^254^, ^255^, ^256^, ^257^ For example, a null mutant of the *bantam* gene in *Drosophila* implicated this miRNA in translational repression of the pro‐apoptotic gene *hid* and control of cell proliferation, cell death,^252^ and later in the maintenance of germ line stem cells.^8^ Similarly, analysis of null alleles was used to demonstrate that *Drosophila miR‐9a* participates in regulating various processes such as sensory organ specification,^258^ or apoptosis in the developing wing by regulating *LIM‐only* (*dLMO)*.^253^ Targeted deletion of the *miR‐124* hairpin implicated this highly conserved neuronal‐enriched miRNA in a variety of developmental and pathological processes. For instance, it was shown that *miR‐124* almost completely represses *anachronism* (*ana*) in the *Drosophila* brain neuroblast lineage, thereby controlling neuronal differentiation^7^ and the number of adult neurons.^255^ Furthermore, subsequent studies revealed that *miR‐124* also plays an important role in neurodegeneration, lifespan, and aging.^256^, ^257^ In vertebrates, one of the first miRNA mutant phenotypes revealed the cardiac growth function of *miR‐1* and *miR‐208*.^259^ Interestingly, under normal homeostasis, without subjecting the heart muscle to physical challenge, *miR‐208* mutants displayed no overt phenotypes. However, upon increasing the afterload on the heart muscle, *miR‐208* null mice completely lacked any signs of hypertropism and fibrosis, both anatomical hallmarks of wild‐type hearts exposed to thoracic aortic banding.^259^

Crossing the boundary of serendipitous or opportunistic discovery of individual miRNA functions required the development of high‐throughput LOF resources. The first miRome‐wide mutant collection was created in *C. elegans* by combined chemical (EMS, DEB) and physical (UV‐TMP) mutagenesis, followed by isolation of short miRNA deletions using an ingenious ‘poison’ primer method.^142^ This allowed an unprecedented level of analysis with regard to the global impact of miRNA regulation on organismal development, viability, and innate adult behavior. Surprisingly, this study revealed that null mutations in most *C. elegans* miRNAs (95 individual miRNA were tested), are viable and do not exhibit any gross morphological phenotypes.^142^ Subsequently, mutagenesis of the majority or all miRNAs within 15 seed‐redundant families uncovered dramatic lethal phenotypes in three cases, suggesting that the paucity of phenotypes in the original screen was partly due to functional redundancy.^260^ Interestingly, a parallel study revealed that 25 out of 31 miRNA null mutants presented with a phenotype when tested in a sensitized genetic background, underscoring the propensity of miRNAs to function in combinatorial networks with other gene regulatory systems.^261^

Subsequently, a comprehensive collection of *Drosophila* miRNA mutants covering over 99% of annotated miRNAs (130 genes in total) was generated by targeted HR, and recently screened for developmental and adult phenotypic abnormalities.^262^ In this instance, developmental progression, survival, primordial germ cell number, lifespan, fertility, climbing behavior, and ovary/external morphology, were systematically monitored across the entire collection. Intriguingly and in contrast to the results obtained in *C. elegans*, this comprehensive screen revealed that over 80% of *Drosophila* miRNA mutants scored positive for at least one of the tested phenotypes.^262^ Based on this study, it was also suggested that the abundance of miRNAs only marginally correlated with phenotypic expressivity, and ablation of miRNA clusters did not appear to show any statistically significant increase in the mutant phenotypes under investigation.^262^ However, functional redundancy within seed‐sharing miRNA families has been observed in *Drosophila* as well. For example, the k‐box miRNA family members *miR‐6* and *miR‐11*, appear to act redundantly in regulating apoptosis during embryogenesis, and only their concomitant deletion rendered central nervous system defects.^263^

In vertebrates, the first mouse miRNA knockout resource covering 476 miRNA genes was generated using recombinant mediated cassette exchange (RMCE) targeting vectors.^264^ C57BL/6N germline‐transmissible embryonic stem (ES) cell clones were then produced containing targeted deletions for 392 miRNA genes. Taking advantage of the FRT and F3 sites intrinsic to the RMCE cassette, it was demonstrated that this resource could be effectively used for the generation of both reporter as well as conditional miRNA alleles. Subsequently, a library of miRNA HR vectors targeting 194 conserved mouse miRNAs, as well as the generation of 46 Cre‐lox conditional miRNA knockout mice, was reported.^143^ Underpinning functional redundancy within miRNA families and interdependence with other gene regulatory pathways, the vast majority of the miRNA deletion mutants in this collection appeared to be viable.^143^ These results underscored the importance of employing combinatorial approaches for the investigation of miRNAs both during normal developmental homeostasis, as well as under genetic, physical, or environmental pressure.

The generation of miRNA deletion mutants by classical HR approaches is a laborious and time‐consuming process in almost all model organisms. Therefore, the construction of comprehensive miRome‐wide mutant resources deserves to be regarded as a feat of scientific strength achievement and acknowledged appropriately. However, the recent advent of powerful genome engineering technologies based on *programmable DNA binding proteins* (PDB) and *RNA‐guided endonucleases* (RGENs) of the *clustered, regularly interspaced, short palindromic repeats* (CRISPR/Cas9) family,^265^ is without doubt rapidly changing this perspective. Thus, using both transcription activator‐like effector nucleases (TALENs) and the CRISPR/Cas9 system, rapid single‐step generation of heritable deletions in individual miRNA genes as well as miRNA cluster loci was recently reported in zebrafish embryos.^266^ In a more advanced version of this approach, highly efficient (87%) and specific bi‐allelic knockout of *miR‐21* was achieved in HEK293 cells, by combining TALEN‐mediated genome editing with an HR template containing the RFP‐puromycin selection cassette.^267^ A similar HDR strategy has been adapted to generate *miR‐21* and *miR‐29a* knockouts in a variety of human cell lines by CRISPR/Cas9 engineering.^268^ Interestingly, a systematic analysis of high‐precision alterations using the CRISPR/Cas9 system revealed that discrete sequence alterations in the 5′ region of the *miR‐93* precursor can impair Drosha processing resulting in consequential miRNA knockout.^269^ Finally, taking advantage of efficient high‐throughput strategies for generation of large TALEN‐based libraries, a collection of 540 TALEN pairs targeting 274 high‐confidence human miRNA genomic loci was recently constructed.^270^ To guarantee complete miRNA LOF, each TALEN pair was carefully designed to either target the seed region of the miRNA or the Drosha processing site. Proof of principle studies revealed that using this strategy, miRNA single and double knockout cell lines could be generated as rapid as 2–4 weeks for functional assays.^270^

## miRNA COMPETITVE INHIBITION

The peculiar features that rendered miRNA mutagenesis efforts challenging, as well as their stereotypical mechanism of action, inspired the development of ingenious alternative strategies for interfering with their activity with unprecedented specificity and spatial‐temporal versatility. Most of these complementary approaches relied primarily on sequestering miRNAs away from their endogenous targets by supplying an artificial high‐affinity competitive inhibitor. Since these molecules bind to mature miRNAs in the cytoplasm, they can simultaneously block an entire family of seed‐redundant miRNAs, thus revealing LOF phenotypes which otherwise would have been masked by compensation. Furthermore, competitive inhibitors are capable of uncovering phenotypes of lethal miRNA null mutants. A variety of miRNA competitive inhibitors have been developed which differ in their physical–chemical parameters, mechanisms of action, and delivery methods, depending on the purpose and model system for which they are intended. These include chemically modified antisense oligonucleotides (e.g., antagomirs),^271^ microRNA sponges (miR‐SP),^272^, ^273^ and tough decoys (TuD)^274^, ^275^ (Figure 4).

### Antisense Oligonucleotides (antimiRs)

One of the first attempts to inhibit miRNAs *in vivo* via competitive inhibition used unmodified antisense DNA oligonucleotides (anti‐miDNA), which exhibited contiguous complementarity across the entire length of the mature miRNAs.^276^ Following embryo injection of anti‐miDNA against 11 *Drosophila* miRNAs, only two targeting the closely related *miR‐2a* and *miR‐13a* elicited specific developmental defects associated with head and posterior abdominal segments. Although this study did not investigate the mechanistic consequence of anti‐miDNA on the levels of targeted miRNAs, it provided evidence that antisense oligonucleotides could be used to interfere with miRNA activity *in vivo*. A subsequent study measured the impact of modified antisense oligonucleotides (ASO) on the *miR‐21*‐mediated cleavage of an artificial target in HeLa cell extracts.^277^ In this instance, both DNA ASOs containing 2′‐*O*‐methyl (2′‐*O*‐Me) groups on the last three 3′ end nucleotides, and RNA ASO with 2′‐*O*‐Me groups on all nucleotides and a 3′ aminolinker were tested (Figure 4(f)). Interestingly, only 2′‐*O*‐Me RNA ASOs displayed a dose dependent inhibition of miRNA‐mediated target cleavage.^277^ A similar study investigating the mechanism of inhibition using 31 nt‐long 2′‐*O*‐Me RNA oligonucleotides in HeLa cells and *Drosophila* embryo lysates, suggested that ASOs block the miRNA loaded miRISC in a stoichiometric and irreversible manner.^278^ Finally, injection of 2′‐*O*‐Me ASOs against *let‐7* in *C. elegans* was reported to recapitulate LOF mutant phenotypes demonstrating their *in vivo* applicability for miRNA studies.^278^

Subsequently, chemically modified ASOs have been employed as a cost effective alternative to the laborious generation of miRNA mutants in mammals.^271^, ^279^ Since ASOs can be administered as a drug during the lifespan of an animal, they are also useful for circumventing potential lethality associated with loss of miRNA function during development. The first ASOs used for *in vivo* miRNA inhibition in mice were chemically modified approximately 22 nucleotides single stranded RNA analogs called **antagomirs.** 271 The stability of antagomirs was enhanced by the presence of 2′‐*O*‐Me groups on all sugars, and phosphorothioate backbone modifications at the 5′ and 3′ ends. In addition, the 3′ end was conjugated to a cholesterol moiety to promote cellular uptake (Figure 4(e) and (f)). In general, antagomirs are fully complementary to miRNAs, and appear to promote degradation of the targeted miRNA *in vivo*. 271, 280 Intravenous injection of antagomirs in mice revealed that they distribute throughout the body and can effectively silence miRNA activity in various tissues except for the brain. However, effective brain delivery has been achieved by direct injection into the cortex.^280^ Finally, it was reported that antagomirs act in a dose dependent manner and can induce a massive downregulation of the targeted miRNA, which lasts up to three weeks.^271^, ^280^

An alternative strategy for designing anti‐miRNA ASOs with superior stability and affinity exploited the ingenious principles used in locked nucleic acids (**LNA**) chemistry. LNAs are RNA nucleotides containing a ribose that is locked in a conformation favorable for base paring by linking the 2′‐O to the 4′‐C via a methylene bridge. Owing to this modification, oligonucleotides containing LNAs exhibit an increased affinity for Watson‐Crick base pairing resulting in higher melting temperatures. A study comparing *miR‐21* 2′‐*O*‐Me ASOs to mixed LNA/DNA ASOs containing eight centered LNA nucleotides (Figure 4(f)), revealed that both strategies lead to a comparable degree of miRNA silencing in human cancer cells.^281^ To assess the specificity of LNA‐based ASOs, the inhibition of an exogenously delivered *Drosophila bantam* miRNA was tested using a luciferase reporter assay in human HEK293 cells. The *anti‐bantam* LNA ASOs elicited a dose dependent de‐repression of a heterologous reporter, and were also capable of inhibiting endogenous *bantam* miRNA in *Drosophila* KC167 cells, resulting in an increased abundance of its physiological target *hid*.^282^ *Anti‐bantam* LNA ASOs with double or triple mismatches exhibited less efficient de‐repression of the reporter indicating that imperfect complementarity can reduce the specificity of ASOs.

The value of LNA‐based miRNA ASO strategies for *in vivo* applications was elegantly illustrated in mice using a high‐affinity 16 nt long oligonucleotide targeting the 5′ end of *miR‐122*, which contained more than 50% LNA and a complete phosphorothioate backbone^283^ (Figure 4(f)). Systemic delivery of this ASO into the abdominal cavity of mice elicited a dose dependent reduction in plasma cholesterol, de‐repression of putative *miR‐122* liver targets, and no apparent hepatotoxicity. Furthermore, the efficiency of this unconjugated LNA antimiR appeared to be superior compared to cholesterol conjugated antagomirs or antimiRs with only 30% LNA content. Importantly, the same *miR‐122* LNA‐based ASO design was employed to demonstrate for the first time inhibition of miRNA activity in nonhuman primates. Systemic intravenous injection of African green monkeys revealed the same dose‐dependent decrease of plasma cholesterol and *miR‐122* depletion, which lasted for several weeks. The *miR‐122* ASO accumulated in the cytoplasm of hepatocytes, but treated animals displayed no signs of toxicity.^284^ These encouraging results inspired the exploration for therapeutic applications of high‐affinity *antimiR‐122* ASOs in chimpanzee models of chronic Hepatitis C virus (HCV) infection. Intravenous administration over several weeks of an LNA‐based *miR‐122* ASO under a formulation generically termed SPC3649 (miRavirsen) significantly reduced the amount of HCV RNA in the serum without any indication of adaptive mutations in the viral genome that could confer resistance to treatment.^285^ Consequently, SPC3649 became the first anti‐miRNA therapeutic strategy to be evaluated in Phase II clinical trials. Recently, it has been shown that SPC3649 is also able to interact with the stem‐loop of the primary and precursor transcript of *miR‐122*, and thereby can block processing by Drosha or Dicer, respectively.^286^ Finally, to simultaneously silence entire families of miRNAs, short seed‐complementary ASOs called ‘**tiny LNAs**’ have been developed, which comprise exclusively of 8 mer consecutive LNA nucleotides and a complete phosphorothioate backbone^287^ (Figure 4(f)). The efficiency of tiny LNAs in silencing the activity of seed‐related miRNAs and de‐repressing their targets has been demonstrated for *miR‐221*/*222*, *miR‐17*, *miR‐18*, *miR‐19*, and *let‐7* families in PC3, Huh‐7 and HeLa cells in culture. Unconjugated tiny antimiRs antagonizing *miR‐21* or *miR‐122* were also systemically administered in mice through intravenous injection. They appeared to exert long‐term sequestration of targeted miRNAs coincidental with target de‐repression, and distributed broadly in various tissues except for the brain.^287^ Comparative analysis of *antimiR‐122* tiny LNAs and high‐affinity *antimiR‐122* 15mers revealed that both displayed similar effects on liver specific *miR‐122* targets.^287^

Various highly optimized antimiR formulations targeting the entire miRNA complement in several species, can nowadays be readily purchased from companies such as *Ambion*, *Exiqon* or *GE Dharmacon*, and are routinely used as miRNA competitive inhibitors in cell culture experiments.

### miRNA Sponges

Transgenic expression of RNA transcripts engineered to carry multiple high‐affinity MREs provided an attractive alternative to chemically modified ASOs for miRNA competitive inhibition. This versatile strategy was capable of engendering superior spatial‐temporal resolution by virtue of conditional expression, pseudo‐ and serotype specific viral delivery or cell‐type specific promoters, as well as long‐term inhibition of miRNA activity *in vivo* (Figure 4(c)). Based on their propensity to silence miRNAs by sequestering them away from endogenous targets, these promoter‐driven competitive inhibitors have been generically termed miRNA sponges (miR‐SP) (for review see Refs 288, 289). One of the initial reported designs, entailed adenoviral delivery of a ‘decoy’ construct consisting of tandem perfect complementary *miR‐133* target sites cloned in the 3′UTR of an EGFP reporter gene, in mouse cardiomyocytes and adult mice.^290^ In this instance, the decoy construct was able to suppress *miR‐133* activity *in vivo*, underpinning its role in regulating cardiac hypertrophy.^290^

However, the first systematic study on the design and efficacy of miRNA sponges was reported later in the same year from the Sharp lab.^272^ This seminal study investigated several design principles such as, promoter class, degree of complementarity to cognate miRNA, number of concatenated miR‐SPs copies, activity on seed‐redundant miRNAs, and compared the results with commonly used synthetic ASOs. Expression of miR‐SPs from both RNA polymerase II (Pol II) and RNA polymerase III (Pol III) promoters (CMV and U6, respectively) resulted in comparable levels of miRNA inhibition in HEK293 cells. However, the mechanism underlying Pol‐III‐driven expression of miR‐SPs was puzzling, since U6 transcripts are not efficiently exported from the nucleus and therefore are unlikely to encounter mature miRNAs in the cytoplasm. In terms of design, constructs comprising of 4–7 miR‐SPs with a mismatch bulge at positions 9–12 of the miRNA preventing Ago2‐mediated slicing, elicited stronger miRNA inhibitory effects compared to sponges carrying two perfect complementary target sites (previous design). This result could be attributed presumably to both the higher number of repetitive miR‐SPs as well as the increased stability and affinity of the bulge design. However, under the conditions of strong Pol II‐driven plasmid expression, the correlation between sponge copy number and potency of inhibition seemed to rapidly saturate at approximately six concatenated miR‐SP sites. In contrast, the situation appeared to be different for stably integrated transgenic miR‐SPs where the magnitude of inhibition appeared to scale with sponge copy number, as it was later also reported in *Drosophila* and other species.^272^, ^273^, ^291^ A head‐to‐head comparison revealed that Pol II‐driven miR‐SPs outperformed *2′‐O‐*methyl antagomirs for all miRNAs tested, and elicited at least equally potent target de‐repression compared to LNA ASOs.^272^ Finally, experiments in HeLa cells using reporter constructs uncovered the exquisite seed specificity of miR‐SPs, as well as their versatility in silencing multiple miRNAs belonging to the same seed‐related family.^272^

Inspired by this design, subsequent studies have evolved this concept to accomplish stable expression of miRNA competitive inhibitors by genomic integration *in vivo*. For example, overexpression of miRNA target (miRT) sequences from lentiviral vectors was employed to establish a strategy for stable miRNA knockdown in cultured cells and in the mouse hematopoietic niche.^292^ This study confirmed the superior effectiveness of bulge miRT sites compared to perfect complementary sites, and revealed a dose‐dependent increase of miRNA silencing potential by increasing the number of miRT sequences from four to eight copies per construct. Importantly, a detailed investigation of miRNA knockdown efficacy using powerful detection assays and a panel of miRNAs, cell types, miRT design parameters, and Pol II promoter types, revealed that a high dose of miRT expression is required for maximum repression of miRNAs in primary cells. Thus, reaching a saturation miRNA repression threshold often required a high number of genomic integrated miRT copies, or highly optimized miRT design. Interestingly, although the maximum repressive threshold varied for each miRNA and in most cases appeared to scale with the levels of miRT expression, it did not strictly correlate with the endogenous miRNA levels.^292^

The first account of genetically encoded conditional miRNA sponges enabling spatial‐temporal investigation of miRNAs at virtually any stage of organismal life, was reported in *Drosophila*.^273^ In this case, miR‐SPs consisting of ten concatenated bulged miRNA binding sites were placed in the 3′UTR of a UAS‐driven reporter gene, and transgenic animals carrying these constructs were generated by transposon‐mediated genomic integration. Combining the resulting UAS‐miR‐SP transgenic lines with tissue‐specific Gal4 drivers allowed targeted expression of these specific miRNA competitive inhibitors in a variety of tissues during development and adult life. Importantly, demonstrating their capacity to specifically repress miRNA activity *in vivo*, transgenic miR‐SPs were able to recapitulate a variety of hypomorphic and null miRNA mutant phenotypes, albeit in some cases engendering only partial penetrance or expressivity. Experiments employing various developmental paradigms revealed the exquisite versatility of this technology from defining tissue‐specific miRNA LOF phenotypes to uncovering complex genetic interactions between miRNAs and other genes or discriminating spatially regulated miRNA targets.^273^, ^293^, ^294^, ^295^, ^296^, ^297^, ^298^, ^299^ Since transgenic miR‐SPs are deployed using the binary Gal4‐UAS modular expression system, they provided an extremely versatile platform for developing comprehensive resources enabling high‐throughput investigation of post‐embryonic tissue‐specific miRNA functions. Indeed, a recent study reported the first transgenic collection of conditional miR‐SP lines targeting 141 high‐confidence *Drosophila* miRNAs.^291^ In this instance, to boost transgene expression and avoid epigenetic positional effects, second generation miR‐SPs comprising of 20 imperfect miRNA binding sites (9–12 bulge) were flanked by *gypsy* insulators and landed in specific genomic loci using the *phiC31* directed integration system.^300^ Furthermore, to maximize the levels of miR‐SP expression and their versatility for genetic interaction studies, independent miR‐SP transgenic lines were generated on both the second and third autosomes for each miRNA (282 lines in total). Highlighting the potential of this resource for the discovery of novel tissue‐specific miRNA functions, a targeted screen for miRNAs underlying the structure and function of the adult indirect flight muscle uncovered 14 miRNAs (24% of all muscle expressed miRNAs), which appeared to be essential for maintaining muscle homeostasis.^291^

The original pioneering studies describing the design and versatility of miRNA sponges^272^, ^273^, ^292^ inspired the adaptation of this platform to various experimental paradigms and model systems. Consequently, the conceptual framework of this technology became one of the methods of choice in the field for the discovery of miRNA functions in complex biological systems.^293^, ^298^, ^299^, ^301^, ^302^, ^303^, ^304^, ^305^ In addition, recent studies refined and optimized the original design, thus improving the efficiency and specificity of miRNA sponges.^306^ Finally, the relatively challenging generation of miR‐SP constructs rooted in their repetitive nature, prompted the development of alternative flexible strategies that now allow rapid assembly of concatenated miR‐SP copies using a single directional ligation approach.^307^, ^308^

### Tough Decoys

By manipulating the RNA secondary structure and the delivery system of promoter‐driven competitive inhibitors, an improved design was developed which appeared to elicit superior miRNA repressive activity, presumably due to increased stability and more robust expression.^274^ The defining features of this new type of miRNA decoys were the folding of miRNA binding sites (with perfect or bulged complementarity) into a stem‐loop secondary structure, and the expression of the decoy cassette from strong Pol‐III promoters. The unique stem‐loop design served both to impart some protection of the uncapped/polyA(−) decoy RNA against RNases and miRISC‐mediated degradation, as well as to ensure efficient nuclear export of these transcripts via the Exportin‐5 pathway. Rooted in this conceptual design, the original study used a systematic iterative approach to refine the efficiency and long‐lasting activity of these RNA decoys for silencing miRNAs in various cell types. By varying the number, complementarity and geometry of the MRE, as well as the position and number of surrounding stem structures, a highly optimized design was obtained, which was termed tough decoy (TuD). The TuD architecture consists of an 18 bp stem followed by a loop containing two antiparallel MREs on opposite strands, and a second stem closed by a small terminal loop (Figure 4(d)). Both MREs are flanked by three nucleotide long linker sequences and contain a four nucleotide long insertion between position 10 and 11 to prevent miRISC‐mediated cleavage. In some instances, it was proposed that potential base pairing between the two antiparallel MREs might prevent efficient sequestration of the cognate miRNA. Indeed, when the two MREs displayed substantial complementarity, sequence alterations by point mutations or longer insertions (up to 4 nt) were reported to increase TuD‐mediated repression of miRNA activity. Using reporter assays in PA‐1 and HCT‐116 cells it was shown that the TuD design is a more potent inhibitor of endogenous *miR‐21* than conventional LNA‐based anti‐miRs or equivalent miRNA sponges. Finally, lentiviral TuD transduced cells were reported to exhibit long‐lasting suppression of miRNA activity for more than one month.^274^

To explore conceptually the potential of TuDs for therapeutic applications, which requires exogenous delivery of low molecular weight compounds, a synthetic version was reported termed S‐TuD.^309^ Briefly, S‐TuDs consist of two 2′‐*O*‐methyl modified oligonucleotides, which when annealed resemble the secondary structure of a TuD lacking the terminal loop. When compared to conventional 2′‐*O*‐methyl ASOs this design proved to be more efficient in repressing endogenous *miR‐21* activity in HCT‐116 cells. Moreover, S‐TuD targeting individual members of several different miRNA families showed high specificity suggesting that the 3′ complementarity outside the seed plays an important role in S‐TuD‐mediated silencing. A single transfection of S‐*TuD‐miR‐200c* was able to repress its target miRNA for approximately seven days (corresponding to ~7 cell cycles) in HCT‐116 cells, and resulted in significant upregulation of the *miR‐200c* target ZEB1.^309^

A study comparing several miRNA competitive inhibitor designs, including Pol‐II and Pol‐III miR‐SPs containing seven MREs, a Pol‐III (U6) transcribed TuD, and a U6 miRZIP, found that only the TuD design efficiently silenced the highly abundant *miR‐122* in HuH‐7 cells, as revealed by a reporter assay.^275^ The same anti‐*miR‐122 TuD* was subsequently used for *in vivo* studies in mice. To avoid potential complications associated with lentiviral integration and promote liver transduction, the TuD was encoded into a recombinant adeno‐associated virus (rAAV) packaged as self‐complementary genomes into the AAV9 capsid (scAAV9). Intravenous delivery into mice led to predominant infection of hepatocytes resulting in specific downregulation of *miR‐122* and upregulation of endogenous *miR‐122* targets in the liver. The same treatment was also able to induce a long‐lasting decrease of cholesterol in the serum (>30%). This effect was first noticeable 2 weeks after injection and cholesterol levels remained low over the 25 weeks period of the study. Additionally, sequencing of miRNAs from livers revealed that TuD treatment increased the presence of nontemplate adenosines at the 3′ end of *miR‐122*. Furthermore, this was accompanied by a decrease in mature 23 nt *miR‐122* levels, and a concomitant enrichment of 18–20 nt long *miR‐122* isoforms, suggesting that TuDs promote miRNA degradation *via* the tailing and trimming pathway.^275^

To identify the most potent miRNA competitive inhibitor system, a recent study compared seven different previously published designs.^310^ All inhibitors were expressed from lentiviral vectors under the control of the Pol‐III H1 promoter and their efficiency was assessed using a dual‐luciferase reporter assay. The vectors were either transfected or delivered as active viruses to HEK293T cells. In all experiments, TuDs showed the most potent inhibition on both *miR‐16* and *miR‐203*. To increase the versatility of TuDs, the stem‐loop structure was cloned downstream of a GFP reporter and expressed under the control of a Pol‐II promoter (Figure 4(d)). The TuD inhibitory activity was then tested on several miRNAs and compared to Pol‐II‐driven miRNA sponges. In each case, the TuD design displayed equal or stronger repression of the cognate miRNA demonstrating the feasibility of this approach.^310^

Similar to miRNA sponges, it was recently reported that clustering tandem repeats of up to four TuD hairpins in the 3′UTR of a GFP reporter significantly enhanced the suppression of several miRNAs in HEK293 cells, as revealed by a luciferase reporter system.^311^ To establish the feasibility of simultaneously repressing the activity of unrelated miRNAs, dual‐targeting single TuD constructs carrying antiparallel binding sites for heterologous miRNA pairs were generated. Interestingly, this dual‐targeting design resulted in effective repression of nearly all targeted miRNAs (5 out of 6), and in some cases (*miR‐16*/*miR‐21* pair) the efficiency of suppressing each miRNA was even higher than that of the corresponding single TuD containing two identical MREs. Finally, merging these two conceptual frameworks enabled the concurrent suppression of six unrelated miRNA (*miR‐143*, *miR‐145*, *miR‐146a*, *miR‐203*, *miR‐16*, and *miR‐21*) by a single Pol II‐driven clustered dual‐targeting TuD array.^311^

A convenient lentiviral plasmid collection encoding 1923 human miRNA TuDs and 1214 mouse miRNA TuDs, as well as the ability to order custom design constructs, are now being offered by Sigma^®^ Life Science.

## FUNCTIONAL miRNA‐TARGET INTERACTION DISCOVERY

Perhaps the most crucial phase in deciphering the mechanisms underlying miRNA‐mediate control of biological functions is the identification of primary miRNA targets that are physiologically regulated at post‐transcriptional level. The technologies described so far have had a tremendous impact on our ability to identify direct miRNA–target binding events as well as uncover miRNA‐regulated biological processes. However, defining physiologically relevant functional miRNA–target interactions posed another experimental challenge, which required the development of yet another set of technologies (summarized in Table 4).

### Reporter Assays

**Figure 5:**
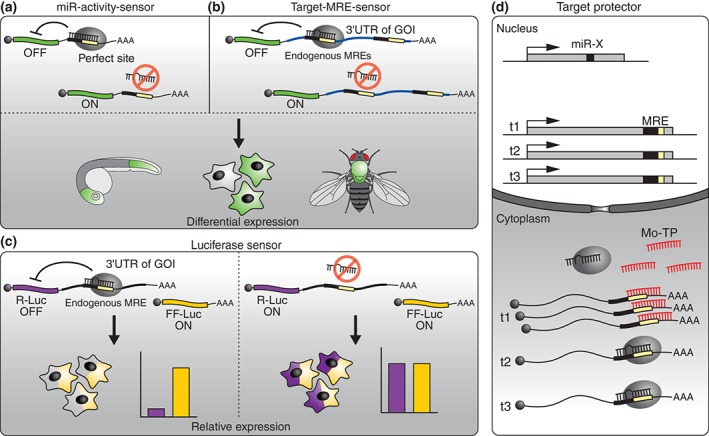
Strategies for discovery and validation of functional miRNA–target interactions. (a) *miRNA‐activity‐sensors* consist of a reporter construct (GFP) appended with a perfect target site for a miRNA of interest. In the presence of an active cognate miRNA, RNAi mediated slicing of the MRE results in loss of reporter expression. In the absence of the miRNA or in the presence of a control sensor with mutant MRE, expression of the reporter gene is detected. GFP‐based sensors allow spatial‐temporal detection of miRNA activity *in vivo*. (b) *Target‐MRE‐sensors* function on a similar principle as miRNA‐activity‐sensors, except that in this case an endogenous 3′UTR (or another part of the gene) containing a putative MRE of interest is fused to a reporter gene, and can be used to infer endogenous regulation of a candidate MRE in an intact organism or in cells in culture. (c) *Luciferase‐based sensors* provide a more quantitative readout of miRNA activity by measuring the relative expression of two distinct reporters. The 3′UTR of a gene of interest is cloned downstream of Renilla luciferase and codelivered to cells together with a control nontargeted Firefly luciferase construct. The ratio between the two reporters is used to quantitatively assess miRNA‐mediated repression of the MRE‐bearing sensor. (d) *Target protectors (TPs)* provide a direct *in vivo* approach for interfering with specific miRNA–target interactions. TPs are complementary with the seed region of a candidate MRE and the sequence immediately adjacent, which increases their specificity and decreases off‐target events. MRE, miRNA response element; GOI, gene of interest; R‐Luc, Renilla luciferase; FF, Firefly luciferase; AAA, poly‐A tail; Mo‐TP, morpholino target protector.

Arguably, the most commonly used experimental strategies for the discovery and validation of functional miRNA targets are the *sensor* or *reporter* systems. These assays were originally designed for monitoring miRNA activity *in vivo* and have been successfully used for this purpose in a variety of biological settings, model systems and organisms.^8^, ^53^, ^252^, ^312^, ^313^ Conceptually, **miRNA‐activity‐sensors** consist of a reporter gene such as luciferase or EGFP, which encodes in the 3′UTR one or more perfect complementary miRNA binding sites (MBS) (Figure 5(a)). They function on the simple assumption that when expressed in cells or intact organisms, the presence of a cognate miRNA will direct Ago‐mediated binding and degradation of the sensor transcript resulting in a quantifiable decrease in reporter output. Since this is a negative detection system, an essential prerequisite for these experiments is parallel evaluation of a control reporter, which either encodes a scramble site or contains mutations in the seed‐match (2–8) of the MBS.^314^ A recent study reported the construction of a large‐scale library of 291 miRNA sensors comprising of five perfect complementary or bulge miRNA target sites placed in tandem in the 3′UTR of EGFP in a lentiviral vector.^110^ Combined with a complementary library of tough‐decoy inhibitors targeting the same miRNAs, and an ingenious multiplex approach for parallel activity profiling (**Sensor‐seq**), this study assessed the impact of miRNA concentration on silencing activity in mammalian cells. Under these experimental conditions, only the top 40% most abundant miRNA covered by this collection displayed silencing activity, and intriguingly, some of the highest expressed miRNAs elicited relatively modest sensor repression.^110^ Negative detection strategies were recently complemented by a novel design, which generates a positive readout of miRNA activity. This system uses a synthetic RNA oligonucleotide that displays perfect complementary to a miRNA of interest, and contains a paired intra‐molecular quencher and reporter group.^315^ Upon interaction with a cellular miRNA, Ago2‐mediated slicing physically separates the reporter from the quencher, and the ensuing signal can be detected by an appropriate system. While this strategy provides an ingenious platform for parallel quantification of multiple miRNAs, it is only applicable to cell culture systems.

Although perfect complementary MBS sensors represent an extremely powerful tool for monitoring the spatial and temporal distribution of miRNA activity, they are not designed to uncover functional miRNA–target interactions. However, using the same conceptual design, the 3′UTR of a gene or a region flaking and endogenous MRE of interest can be fused to a fluorescent reporter, providing an opportunity to assess potential miRNA‐mediated control of candidate target genes expression, by evaluating reporter output.^53^, ^250^, ^252^, ^312^ To isolate the activity of a specific MRE of interest, a reporter construct containing disruptive mutations in the predicted MRE seed region or completely lacking the MRE of interest is analyzed in parallel in the same cells or tissues. Both GFP and luciferase fluorescent proteins have been used in the design of such **target‐MRE‐sensor** systems (Figure 5(b)). GFP provides a convenient visual detection system for *in vivo* tissue‐specific studies, while cell culture‐based luciferase systems allow more accurate measurements and enable high‐throughput assays. *Target‐MRE‐sensors* can be deployed to cells or intact organisms in the form of plasmid DNA vectors, *in vitro* transcribed RNA constructs, or transgenically expressed for genomic loci. The mutant and wild‐type 3′UTR sensors can also be simultaneously expressed ideally from bidirectional promoters, either by using two fluorescent proteins (GFP and RFP) or the dual *firefly*/*Renilla* luciferase system. Routed in their ubiquitous expression, *in vitro* transcribed RNA and transgenically encoded GFP‐3′UTR sensors have been successfully used to infer endogenous cell or tissue‐specific miRNA–target regulatory axes in *Drosophila,* zebrafish, and other species.^53^, ^252^, ^301^, ^316^ Conversely, luciferase‐based 3′UTR‐sensors have been extensively employed to study miRNA–target regulation in cultured cells, where they usually revealed more subtle tuning interactions (Figure 5(c)).

This approach can also be used in a heterologous assay where both the 3′UTR sensor carrying a predicted MRE of interest and the cognate miRNA are exogenously delivered to naïve cells.^125^ Since these experiments are performed in cultured cells, they are amenable to high‐throughput multiplex interrogation of miRNA–target interactions. Furthermore, due to their inherent simplicity these assays can be used to study the anatomy of miRNA–target interactions at single‐nucleotide resolution. Although this strategy provides an attractive simple alternative to more demanding *in vivo* experiments in intact organisms, it has several caveats that limit its utility to only predicting plausible miRNA–target functional interactions, rather than identifying *bona fide* physiologically relevant MREs. It is well established that miRNA‐mediated regulation under homeostasis is contingent upon and modulated by the balance between available miRNA copies and the abundance of their cognate MREs. Under this premise, the main limitation of heterologous sensor assays is that the analysis is performed at nonphysiological stoichiometry, since both the miRNA and the 3′UTR‐sensor are overexpressed from exogenous promoters. Furthermore, in certain cases these assays have generated conflicting results, which questioned their intrinsic reliability. For example, two independent studies employing what appeared to be a similar *unpaired*‐3′UTR luciferase sensor system in *Drosophila* S2 cells, reported divergent results regarding the response to *miR‐279* overexpression.^302^, ^317^

Inherent to their expression from artificial promoters or ectopic delivery, most miRNA sensor systems do not recapitulate the endogenous transcript level of MRE‐bearing 3′UTRs. In addition, these chimeric constructs may disrupt contextual features playing important roles in miRNA‐mediated silencing, such as RNA secondary structures, RBP occupancy, and alternative poly‐adenylation.^30^, ^119^, ^318^ Therefore, although a valuable experimental tool for miRNA studies, sensor assays have limited ability to accurately infer the physiological relevance of MREs, and cannot be used to assess the phenotypic consequences of blocking endogenous MREs *in vivo*. Consequently, these assays should always be used in conjunction with complementary strategies capable of providing direct experimental evidence regarding functional miRNA–target regulatory interactions.

### Target Protectors

A significant step forward in the ability to discover and characterize functional MREs *in vivo* was marked by the development of target protector (TP) oligonucleotides^319^, ^320^, ^321^ (Figure 5(d)). In essence, TPs are antisense oligonucleotides containing either a modified morpholino backbone or LNA nucleotides, which increase their cellular stability, target binding affinity, and prohibit their incorporation into the RISC complex. In contrast to miRNA competitive inhibitors, TPs are typically complementary to only the seed region of a putative MRE, and a stretch of nucleotides immediately adjacent (downstream) to the MRE of interest. Therefore, instead of globally interfering with miRNA activity, and implicitly perturbing all its endogenous primary and secondary targets, TPs can be used to dissect specific miRNA–target interactions in their endogenous cellular context (Figure 5(d)). As such, TPs represent the first account of a technology amenable to deciphering the functional and phenotypic consequences of interfering with primary MREs *in vivo*. The design, construction, and experimental strategies underlying the use of morpholino‐based TPs have been extensively described in a comprehensive protocol report published by the Giraldez group.^320^ Since their original description, TPs have been successfully employed to study miRNA–target interactions in a variety of experimental systems and model organisms, including zebrafish, *Drosophila*, and mammalian cells.^319^, ^321^, ^322^, ^323^ For instance, in zebrafish morpholino‐based TPs enabled the discovery of an exquisite role of *miR‐430* in modulating Nodal signaling by tuning the expression of *squint* and *lefty* via specific MREs.^319^ In a similar fashion, it was discovered that *miR‐430* also controls the accuracy of germ cell migration in zebrafish embryos, by directly targeting *sdf1a* and *cxcr7* mRNAs.^321^ In *Drosophila*, transgenic expression of a target protector directed at a putative *let‐7* MRE in the *dp* mRNA 3′UTR was used to validate the role of this miRNA–target axis in LRRK2 pathogenesis.^322^

Although an extremely promising approach, TPs do have several potential limitations that may restrict their implementation and possibly render erroneous results. One disadvantage of chemically modified TPs is that they require administration by injection or transfection, which implicitly limits their range of applications to systems amenable to such delivery methods. Furthermore, their activity span is restricted to only approximately 5 days post‐delivery.^320^ However, attempts to transgenically express unmodified TPs from genomic loci have shown promising results in *Drosophila* and mice, providing a potential solution for expanding their usage.^322^, ^324^ The integration in the TP design of extended complementarity to the sequence surrounding the MRE of interest, together with the modified backbone, confers increased on target affinity and decreased off‐target effects. However, the complementarity to the seed region could still enable binding to other seed‐related MREs, especially if the flanking sequences are similar to that of the targeted MRE. Finally, it has been noted that morpholino oligonucleotide injections in zebrafish do not always phenocopy null mutants, suggesting they educe toxic or off‐target effects.^325^

Nonetheless, TPs provide a powerful tool for interfering with specific miRNA–target interactions, and can be commercially acquired from Qiagen (miScript) and Exiqon (miRNA Target Site Blocker), the latter containing LNA nucleotides for improved affinity and reduced background effects.

### The Next Frontier—Genome Engineering MREs

Considering the impressive pace in the evolution of genome editing tools and their recent influence on technology development across life sciences, it was only a question of time until they would impact miRNA functional studies. Thus, in the quest for establishing a strategy that could allow unequivocal functional interrogation of primary miRNA targets, several recent studies have turned toward two game changing technologies: TALENs and the CRISPR/Cas9 system. Conceptually, all these approaches endeavored to directly mutagenize single or multiple MRE genomic loci, as a new experimental paradigm for the discovery and characterization of functional miRNA–target regulatory axes. Initially, multiplex CRISPR/Cas9 genome engineering was used to generate unspecific large deletions within the 3′UTR of the FIH1 gene in NSCLC lung cancer cells.^326^ In this case, cells were simultaneously cotransfected with a Cas9‐expressing plasmid and twelve sgRNAs targeting various positions in the FIH1 3′UTR. Following clonal selection, cell lines carrying mono and bi‐allelic deletion of various sizes were recovered, with the largest homozygous deletion covering 3.77 kb of the FIH1 3′UTR which removed most of the predicted MREs. Further analysis using this clonal line revealed a significant increase in FIH1 expression and concomitant reduction in tumor growth, which were comparable to the effects observed in *Dicer* mutant cells.

The first systematic account of adapting programmable genome engineering strategies to enable high‐resolution mapping of functional MREs and assess their activity in intact biological systems, was reported in the same year.^327^ In this case, both TALE nucleases and the CRISPR/Cas9 system were used to genetically alter predicted MREs in zebrafish, *Drosophila* and human cells in culture. Genome editing using these technologies relies on the initial generation of DNA double‐stranded breaks (DSBs) at specific sites in the genome and their subsequent repair using one of two DNA repair pathways. The error‐prone nonhomologous end‐joining pathway (NHEJ) can generate high‐frequency insertion or deletion mutations (indels), which if induced in close proximity or overlapping a miRNA target site, could result in loss of MRE function. Alternatively, if an exogenous ‘donor template’ is provided, the homology‐directed repair (HDR) pathway can be exploited to incorporate or remove specific sequences at precise genomic locations. Experiments employing TALE nucleases in zebrafish embryos showed that removing a predicted *miR‐430* MRE in the *lefty2* 3′UTR result in target upregulation and a dramatic phenotype in the embryo, in agreement with and validating previous studies using *miR‐430‐lefty2* TPs.^319^, ^327^ Notably, owing to the high frequency of indel generation, it was proposed that such MRE functional studies could be carried out in transient assays, without the necessity of establishing clonal fish lines. To assess the applicability of this conceptual framework to functional analysis of MREs during development and adult lifespan, the CRISPR/Cas9 system was employed to edit MREs in the *Drosophila* germline. Analysis of a predicted *bantam* target site in *enabled* 3′UTR revealed that although this MRE could mediate target repression at nonphysiological miRNA levels, in contrast to expectations from earlier sensor studies^301^ it did not appear to be repressed by *bantam* under normal homeostasis.^327^ To assess the physiological relevance of MREs in transiently transfected cells in culture, a novel CRISPR/Cas9‐based HDR strategy was developed, which takes advantage of a pair of single stranded DNA barcoded repair templates. Experiments in HEK293T cells and *Drosophila* S2R+ cells revealed that this approach was highly efficient and could be applied to study the activity of both canonical and noncanonical MREs.^327^ Finally, an algorithm and online resource was developed which facilitates rapid prediction of CRISPR target sites and guide RNA design of all putative MREs in the *C. elegans*, *Drosophila*, mouse, rat, and human genomes (miR‐CRISPR).^327^

CRISPR/Cas9 MRE engineering was also recently used to investigate the molecular basis of *let‐7*‐mediated vulval bursting phenotype in *C. elegans*.^328^ Surprisingly, this study revealed that removing approximately 450 bp from the *lin‐41* 3′UTR which includes two predicted *let‐7* MREs, is sufficient to cause a vulval bursting phenotype similar to *let‐7* LOF mutants. Furthermore, introduction of precise mismatches (G:U wobbles) in each of these two *lin‐41* MREs *via* CRISPR/Cas9 HDR, resulted in a partial upregulation of LIN‐41 expression, resembling a *let‐7* hypomorphic phenotype. In addition to underscoring the limitations of previous indirect functional MRE assays, this study further highlighted the potential of genome engineering in establishing the physiological relevance of a miRNA–target interaction with regard to a particular biological process.

**Figure 6:**
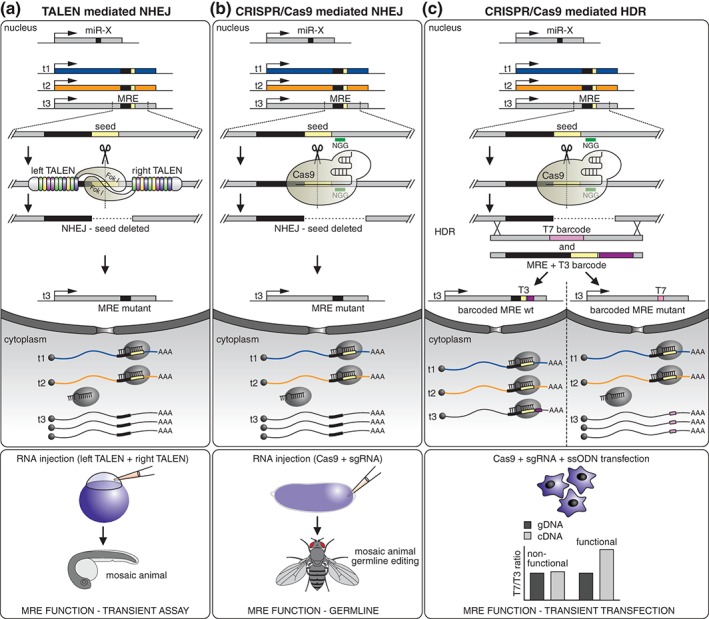
Discovery and characterization of functional MREs by genome engineering. (a) Injection of *in vitro* transcribed RNA encoding a TALEN pair targeting approximately 20 bp flanking a predicted MRE site into single cell zebrafish embryos can be used to generate NHEJ‐mediated precise deletions of the MRE seed sequence. High efficiency editing events enable direct functional analysis of miRNA–target axes in mosaic animals without the necessity of establishing clonal lines. (b) Taking advantage of the simplicity and superior versatility of CRISPR/Cas9 genome engineering strategies, germline‐transmissible precise MRE deletions can be rapidly generated by injection of the Cas9 nuclease and a synthetic guide RNA (sgRNA) targeting the MRE of interest in *Drosophila* syncytial blastoderm embryos. Homozygous MRE mutant lines can then be used to study the consequence of interfering with a specific miRNA–target axis during development and adult life of an organism. (c) CRISPR/Cas9 genome engineering can also be adapted to assess the endogenous activity of specific MREs in human cells without the necessity of establishing clonal lines. This system relies on altering an MRE of interest by CRISPR‐mediated homology‐directed repair (HDR) using two user‐defined ssDNA oligonucleotide repair templates. The first template deletes the MRE and replaces it with a T7 ‘barcode’, while the second maintains the MRE and adds a T3 ‘barcode’. This allows the activity of the MRE to be analyzed in a heterogeneous mixture of cells by comparing the levels of mRNA containing the T7 ‘barcode’ where the MRE has been deleted, to the T3 ‘barcode’, where the MRE remains intact. This can be accurately quantified by measuring the mRNA levels (cDNA) normalized for HDR integration efficiency to genomic DNA (gDNA) from the same sample. TALEN, Transcription activator‐like effector nuclease; CRISPR/Cas9, clustered regularly interspaced short palindromic repeats (CRISPR)‐associated nuclease (Cas9); MRE, miRNA response element; NHEJ, nonhomologous end joining; HDR, homology‐directed repair; FokI, FokI nuclease; ssODN, single stranded DNA oligonucleotides; cDNA, complementary DNA; gDNA, genomic DNA.

It is important to note that all CRISPR/Cas9‐based approaches require relatively minimal time and resources, and can be applied to the ever‐expanding variety of cell culture systems and whole organisms amenable to genome engineering. Furthermore, due to the effortless design and assembly principles of guide RNAs as well as highly efficient DNA cleavage activity,^329^ the CRISPR/Cas9 provides an experimental platform that may enable in the future multiplex functional MRE mutagenesis screens (Figure 6).

## CONCLUSIONS AND OUTLOOK

**Figure 7:**
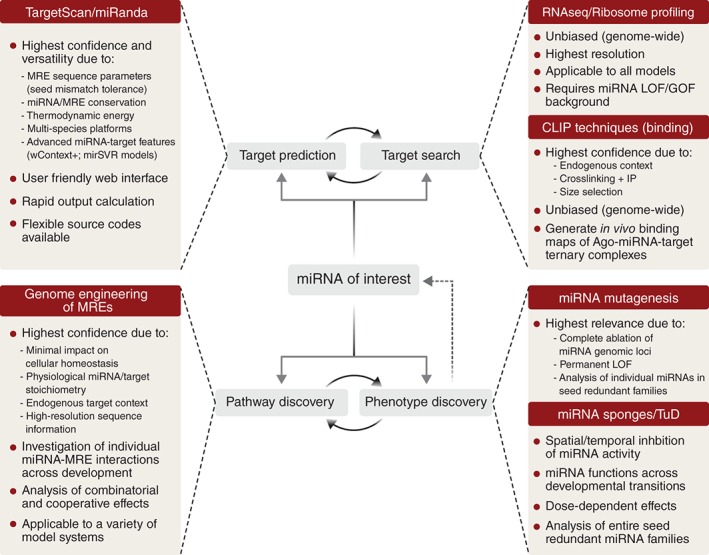
Suggested experimental guideline for systematic interrogation of miRNA functions. A typical miRNA functional study entails a multipronged approach encompassing several layers of analysis. These include but are not limited to: *in silico* target prediction, unbiased *in vivo* interrogation of putative target networks, establishing phenotypic consequences of interfering with miRNA homeostasis (LOF), and discovery of physiologically relevant downstream targets and pathways. However, the order in which each of these phases is implemented can vary, and frequently some analyses can be carried out in parallel or are synergistically reinforcing each other. Similarly, the experimental strategies supporting such a pipeline are also subject to bias. The techniques outlined here reflect our preference regarding the current state‐of‐the‐art strategies underlying each of these steps.

Few biological breakthroughs have gathered the continued attention and interest that miRNAs have engendered since their discovery over 22 years ago. Although the first miRNA reported in *C. elegans* was considered an idiosyncrasy, their subsequent recognition as a new dimension of genome regulation remains arguably one of the most defining landmarks in recent biomedical research. From a bird's eye view perspective, perhaps one of the most distinctive features of this fascinating field of biology is its intertwined evolution with technology development. Progress across all biology exploration is inherently technology driven but only a select few research areas have profited exponentially from the vast technical advances of the last decade. Perhaps atop this list, the study of miRNAs has seen an astounding and above all dependence on and immediate translation of novel technology platforms. From profiling miRNAs across phyla, to deciphering their biogenesis and mechanism of action, to identifying underlying regulatory networks and biological functions, our understanding of miRNAs relied on the development of a sophisticated experimental toolkit. However, this astonishing technological revolution inadvertently increased the experimental landscape complexity, which consequently made it more difficult to choose the appropriate tool for a given analysis. In many instances, this choice relies primarily on the available expertise and resources. Furthermore, miRNA functional studies can be approached from many different angles. Therefore, it is difficult to envision a universally applicable experimental workflow. Solely as a guideline, an example of a systematic miRNA research pipeline is provided in Figure 7. At each stage within this pipeline a variety of techniques can be employed, most of which have been discussed in detail throughout this review. Although inherently each technology has advantages and limitations, as a result of continuous iterative refinements and implementation of novel breakthrough principles, some strategies offer in our opinion superior dynamic range, efficiency, and reliability (Figure 7 boxes). In particular, the evolution of deep sequencing technologies, and CLIP methods, and their adaptation to miRNA research, has occurred at an unparalleled pace and amassed an immense amount of data. These high‐throughput approaches helped shed light on many mechanistic questions underlying miRNA‐mediated post‐transcriptional regulation.

While many tantalizing examples of biological functions have been attributed to single miRNA–target interactions, it is likely that we have only scratched the surface regarding the potential global impact of miRNAs on controlling gene expression. Furthermore, despite the impressive amount of knowledge accumulated over the past two decades, much like a nested doll, with almost every discovery of a miRNA function, breakthrough principle, or regulatory dimension, a new and often unexpected layer of complexity is being uncovered. Perhaps one of the most important unmatched dimensions in miRNA biology is reaching a better understanding of the interface between miRNAs and their targets at the functional level in the endogenous context of a cell. This however turns out to be an extremely complex puzzle and solving it will require both ‘high‐resolution’ *in vivo* interrogation of endogenous MRE environments, as well as a vantage point evaluation of miRNAs from a transcriptome‐wide network perspective.

With regard to the endogenous MRE environment, a major question in the field remains how miRNAs chose their targets amidst hundreds of base‐pairing options displayed by canonical and noncanonical MREs. A recent series of *in vitro* studies using state‐of‐the‐art imaging technologies provided invaluable high‐resolution data regarding the kinetics of miRNA‐mediated Ago2 biding to seed‐bearing MREs. Based on these results, it was proposed that binding occurs in a step‐wise fashion involving initial transient base pairing at nucleotides 2–4 of the miRNA, followed by subsequent stable association when the entire seed (nucleotides 2–8) is matched by complementarity with the target.^17^, ^18^, ^19^ Although this marked a major breakthrough in our understanding of miRNA–target binding at molecular level, the rules governing miRNA target selection and repression *in vivo*, remain enigmatic. Intriguingly, despite the fact that miRNAs are often co‐expressed with a large number of putative targets carrying seemingly identical seed‐sequence MREs, they appear to preferentially regulate only a select few and to various degrees. Some of the parameters underpinning and modulating the propensity of miRNAs to regulate their targets have been proposed, but they are insufficient to establish generalizable predictable models. Another feature that appears to be intrinsic to the MRE environment is mechanistic variability. Thus, while in rare cases the result of miRISC binding to MREs can resemble a binary switch mechanism, most frequently the consequential effect on target levels is fine‐tuning gene expression, often in a cooperative manner with transcription factors. The overt regulatory functions of miRNAs can also vary drastically from shaping developmental transitions, to controlling cellular homeostasis, dampening or elevating the level of intrinsic noise in gene networks, or establishing expression thresholds by controlling spurious transcription.^109^, ^330^ Overall, most of the sequence or context determinants underlying all these mechanistic and functional diversity in miRNA regulation remain to be discovered. Addressing these questions in detail will require the development and adaptation of novel high‐throughput technologies for disrupting MREs with molecular precision, and evaluating in a multiplex fashion the consequences of interfering with whole miRNA target networks *in vivo*. Eventually, deciphering these novel dimensions of miRNA biology will play a central role in understanding their global contributions to developmental transitions, as well as anticipating the broad consequences of manipulating miRNA function for therapeutic intervention.

From a broad cellular perspective, because in many species more than half of the coding transcriptomes are estimated to encode conserved miRNA target sites, each miRNA is predicted to be co‐expressed and interact with hundreds of mRNA targets.^331^ Furthermore, since most genes encode target sites for various miRNAs, it is reasonable to assume that multiple miRNAs may act cooperatively to regulate important targets within a defined cellular or biological context.^26^ Based on these considerations, it has been speculated that under certain circumstances, highly abundant MRE‐bearing RNAs have the potential to sequester or titrate away miRNAs from their other targets, thus eliciting an endogenous ‘sponging’ effect. It has been proposed that this phenomenon might provide an additional regulatory layer of miRNA activity, leading to the formulation of the competitive endogenous RNA (ceRNA) hypothesis.^332^ According to this hypothesis, the activity of a miRNA is influenced by the abundance of its cognate MREs and their crosstalk, which can modulate its relative repression potential.^331^ This mechanism has been first described in plants as a natural mechanism of miRNA inhibition termed ‘target mimicry’.^333^ In animals, the first experimental evidence of the ceRNA phenomenon was reported for the pseudogene *PTENP1*, which appears to control the expression of its protein‐coding tumor‐suppressor counterpart *PTEN* in a miRNA‐dependent manner.^334^ A number of related studies revealed that regulation of *PTEN via* ceRNA crosstalk networks has direct clinical relevance in a variety of cancers, including prostate carcinoma, melanoma and glioblastoma.^335^, ^336^, ^337^, ^338^ Similarly, a recent study proposed that the pseudogene *BRAFP1*, which shares target sites for numerous miRNA families with *BRAF* in both mice and humans, could promote aggressive diffuse large B cell lymphoma by a ceRNA mechanism and consequential upregulation of *BRAF* expression.^339^ A number of other studies revealed that the range of RNA species capable of ceRNA regulatory functions includes pseudogenes, long noncoding RNAs (lncRNAs), and viral RNAs.^332^ The dysregulation of ceRNA crosstalk regulatory networks has also been implicated in neurodegenerative disorders, as it was recently reported for the case of lnc‐SCA7 and *miR‐124* in spinocerebelar ataxia type 7.^340^ Furthermore, virus encoded RNAs or viral RNA genomes can also impact the homeostasis of miRNA networks in infected cells. For example, a recent systems‐level analysis suggested that the HCV RNA genome could function as a ceRNA, and this effect may be linked to the tumorigenic potential of this virus associated with chronic infections.^341^ A unique feature of the HCV is its dependence on the liver expressed *miR‐122*, which binds and stabilizes the 5′UTR of the viral genomic RNA, a mechanism that has been successfully exploited for therapeutic intervention.^13^ Surprisingly however, the recent data revealed that this interaction results in a global and specific de‐repression of *miR‐122* targets in hepatocytes, much like the mechanism proposed for endogenous ceRNAs, which eventually impacts liver homeostasis.^341^ Finally, a new dimension of the ceRNA regulatory crosstalk emerged with the renewed appreciation of endogenous circular RNA transcripts (circRNAs), which are generated from back splicing of primary mRNA transcripts, and appear to be highly prevalent in mammalian cells.^342^ Two independent studies revealed that an unusually stable circRNA encoding as many as 70 conserved target sites for *miR‐7*, acts as a ceRNA decoy by de‐repressing *miR‐7* targets in neuronal tissues.^343^, ^344^

Although the ceRNA crosstalk appears to be a central regulatory mechanism of miRNA activity in all reported studies, its widespread impact on miRNA target networks in primary cells under normal homeostasis remains controversial. A genome‐wide bioinformatics analysis of glioblastoma cells suggested that modulation of miRNA activity through ‘sponging’ could be a relatively widespread phenomenon in physiology and disease.^337^ However, a recent study asserted that oscillations in the abundance of single targets are unlikely to significantly impact miRNA‐mediated regulation through a ceRNA mechanism.^331^ This perspective is supported by the fact that under normal homeostasis any single target transcript, even if expressed at high levels, represents only a small fraction relative to the abundance of all possible cognate target sites of one miRNA. Indeed, taking advantage of a controlled delivery system for one *miR‐122* validated target, this study revealed that in hepatocytes global derepression occurred only when the threshold of ceRNA‐derived target sites approached equimolar concentrations equal to all putative *miR‐122* MREs in physiological or disease states.^331^ All together, these studies suggest that the verdict regarding the universality of the endogenous ceRNA hypothesis still remains to be determined, and future research as well as the development of more refined technologies will be required to provide a definitive answer. Nonetheless, from a technique development standpoint, the concept of competitive inhibition has been successfully harnessed experimentally for a long time, and had a major impact in both basic miRNA research studies as well as in potential therapeutic applications (see ‘Competitive Inhibitors; section in this review).^310^

Although we have undoubtedly learned a great deal about miRNA biogenesis, their mechanisms of action, regulatory functions and roles in development, physiology, and disease, the quest for understanding these tiny giants of gene regulation is far from being completed. Therefore, it is likely that their intriguing nature, fascinating cellular roles, and tremendous therapeutic potential, which have captured the scientific imagination since their discovery two decades ago, will continue to engage the biomedical research community for many years to come.
